# Mutations in the essential outer membrane protein BamA contribute to *Escherichia coli* resistance to the antimicrobial peptide TAT-RasGAP_317-326_

**DOI:** 10.1016/j.jbc.2024.108018

**Published:** 2024-11-26

**Authors:** Maria Georgieva, Filip Stojceski, Fabian Wüthrich, Carole Sosthène, Laura Blanco Pérez, Gianvito Grasso, Nicolas Jacquier

**Affiliations:** 1 Institute of Microbiology, University Hospital and University of Lausanne, Lausanne, Switzerland; 2 Dalle Molle Institute for Artificial Intelligence, IDSIA USI-SUPSI, Lugano, Switzerland

**Keywords:** Gram-negative bacteria, outer membrane, antibiotic resistance, antimicrobial peptide, outer membrane proteins, cell surface

## Abstract

Antimicrobial peptides (AMPs) are promising alternatives to classical antibiotics against antibiotic-resistant pathogens. TAT-RasGAP_317-326_ is an AMP with broad range antibacterial activity, but its mechanism of action is unknown. In this study, we analyzed a strain of *Escherichia coli* with extensive resistance to TAT-RasGAP_317-326_ but not to other AMPs that we obtained after twenty passages during an *in vitro* resistance selection experiment. This strain accumulated four mutations. One of these is a point mutation in *bamA*, which encodes an essential protein involved in the folding and proper insertion of outer membrane proteins. The mutation resulted in a change of charge in a surface-exposed negatively charged loop of the BamA protein. Using CRISPR-Cas9-based targeted mutagenesis, we showed that mutations lowering the negative charge of this loop decreased sensitivity of *E. coli* to TAT-RasGAP_317-326_. *In silico* simulations unveiled the molecular driving forces responsible for the interaction between TAT-RasGAP_317-326_ and BamA. These results indicated that electrostatic interactions, particularly hydrogen bonds, are involved in the stability of the molecular complex, representing a predictive fingerprint of the TAT-RasGAP_317-326_ - BamA interaction strength. Interestingly, BamA activity was only partially affected by TAT-RasGAP_317-326_, indicating that BamA may function as a specific receptor for this AMP. Our results indicate that binding and entry of TAT-RasGAP_317-326_ may involve different mechanisms compared to other AMPs, which is in line with limited cross-resistance observed between different AMPs. This limited cross-resistance is important for the clinical application of AMPs towards drug-resistant pathogens.

Gram-negative bacteria possess an outer membrane (OM) that is a powerful barrier against many antimicrobial agents (1, 2). This membrane is asymmetric, with an inner leaflet composed mainly of phospholipids and an outer leaflet composed of lipopolysaccharides (LPS) (3). The outer membrane is characterized by the presence of integral outer membrane proteins (OMPs) that show a wide variety of functions. OMPs can work as porins and efflux pumps, regulating the transport of compounds through the OM (4). They can also be involved in adhesion, host manipulation, biofilm formation and many other processes that are essential for the Gram-negative bacteria (5, 6). It has been shown that modifications of the OM composition and structure can be the cause of resistance to antimicrobial agents (7). Permeability of the OM can be modified through changes in levels of OMPs, which in turn, can result in reduced uptake of antimicrobial drugs (2). Another mechanism of resistance involves the export of antibacterial agents outside of the bacterial cell *via* OMP efflux pumps (8). Proper OM composition is dependent on the activity of the well characterized Beta-barrel Assembly Machinery (BAM) protein complex which is responsible for catalyzing the folding and insertion of beta-barrel OMPs in the OM (9). Insertion of OMPs is performed by BamA with the help of lipoproteins (BamB, BamC, BamD, and BamE), the chaperone SurA and the protease DegP (9). In *Escherichia coli*, BamA and BamD are essential components of the BAM complex, while the other components are accessory. The exact role of BamB, BamC and BamE is not well understood. SurA is a chaperone that is required for the efficient transport of unfolded OMPs through the periplasm. Finally, DegP is a protease that degrades unfolded OMPs that may accumulate in the periplasm (Fig. 1*A*). This complex, and particularly the essential insertase BamA, were recently highlighted as a new promising target for the development of antimicrobial agents (10, 11, 12).

**Figure 1:**
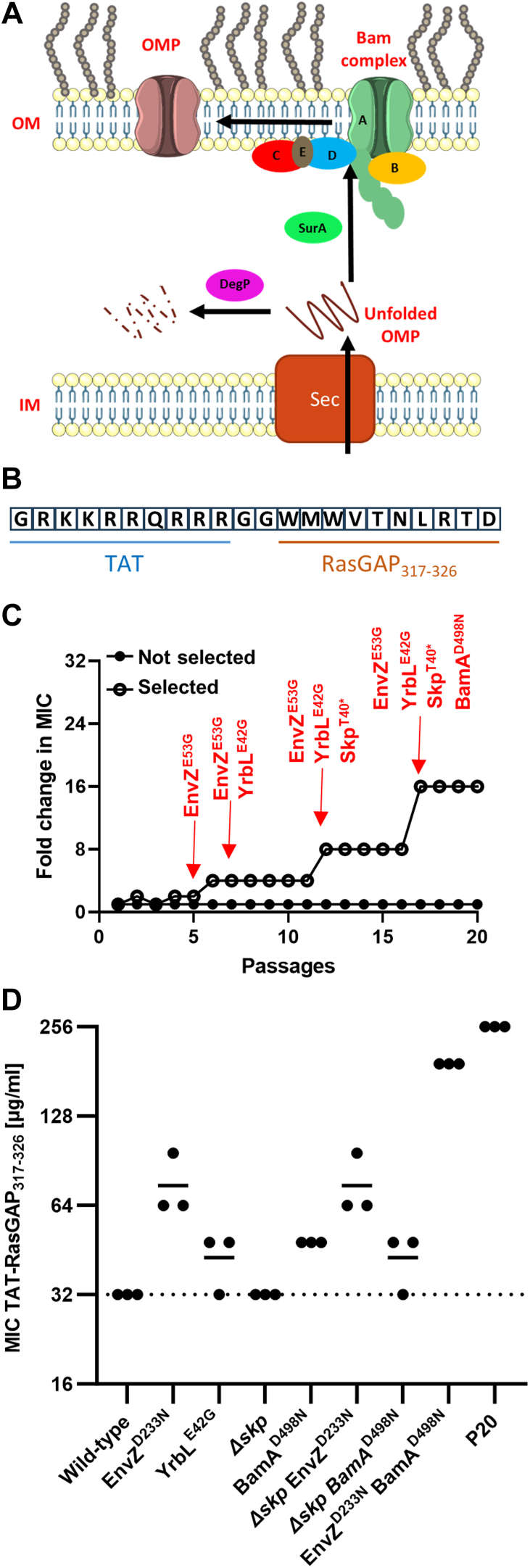
**Sequential acquisition of four mutations confers extensive resistance to the antimicrobial peptide TAT-RasGAP_317-326_ in *E. coli*.** *A*, graphic representation of BAM complex components and their activity for OMP folding and insertion in the OM: BamA, B, C, D, and E, as well as the chaperone SurA and the protease DegP. BamA and BamD are essential. *B*, schematic representation of the amino acid sequence of TAT-RasGAP_317-326_. *C*, acquisition of mutations is linked to increased resistance. *E. coli* MG1655 was incubated with increasing concentrations of TAT-RasGAP_317-326_ for a total of 20 passages. MIC of the peptide on each passage was measured and presented as fold change compared to the parental strain. Whole genome sequencing was performed on the parental and passage 20 (P20) strains. Presence of the four mutations detected was investigated by PCR and Sanger sequencing for all the passages. Passages in which new mutations were detected are highlighted with an arrow. *D*, EnvZ and BamA point mutations have an additive effect on resistance to TAT-RasGAP_317-326_. The indicated point mutants were designed by CRMAGE in wild-type or *∆skp* backgrounds. MICs of TAT-RasGAP_317-326_ on these strains were measured in triplicate and compared to the P20 strain. Dashed line corresponds to the average MIC of the wild-type. Continuous lines show the mean of the triplicates.

Antimicrobial peptides (AMPs) are a naturally occurring component of the innate immune system of a plethora of organisms, from bacteria to humans (13). These usually positively charged peptides possess specific affinity to bacteria through their interaction with the negatively charged bacterial cell envelope, mostly through electrostatic interactions with LPS (14). Modifications of the LPS that reduce its net negative charge are known to cause resistance to certain AMPs (15). For example, modifications of LPS are the main cause of resistance to the clinically used AMP polymyxin B (16).

TAT-RasGAP_317-326_ (Fig. 1*B*) is a 22-amino-acid peptide, first developed as an anticancer agent, capable of entering cancer cells by an auto-induced hyperpolarization of the membrane that induces the formation of transient water pores. Once inside the cytosol, the peptide can bind to inner-leaflet-enriched phospholipids and subsequently cause cell death (17). We determined that, in addition to its anticancer activity, TAT-RasGAP_317-326_ possesses antibacterial properties, as well as potent antibiofilm activity, alone or in combination with classical antibiotics (18, 19, 20, 21). However, the exact antimicrobial mechanism of action (MOA) of this peptide is still not understood.

In this study, we set out to investigate the potential development of resistance against the TAT-RasGAP_317-326_ peptide in *E. coli* by studying a highly resistant isolate we obtained by extended *in vitro* resistance selection. In addition to a mutation targeting the two-component system EnvZ/OmpR that we already described (20), we detected three additional mutations, among which, a point mutation causing an amino-acid change at the surface of BamA—the essential subunit of the β-barrel assembly machinery. This mutation caused resistance to TAT-RasGAP_317-326_ but not to other AMPs. We show here that point mutations that remove negative charges in a highly negatively charged loop at the surface of BamA induced increased resistance to TAT-RasGAP_317-326_. Interestingly, the combination of mutations in both BamA and EnvZ causes the biggest increase in resistance level, indicating that there is a crosstalk between EnvZ and BamA that is required to induce the maximal level of resistance. *In silico* modeling predicted that TAT-RasGAP_317-326_ could directly bind to BamA. However, we could not detect any strong influence of this binding on the activity of BamA, indicating that this protein may function as a receptor for TAT-RasGAP_317-326_.

## Results

### *In vitro* resistance selection to TAT-RasGAP_317-326_ induces the accumulation of four mutations in *E. coli*

In a previous study, we found that exposure of *E. coli* MG1655 to increasing concentrations of the antimicrobial peptide TAT-RasGAP_317-326_ over 20 passages generated a resistant bacterial clone (named P20 from now on) with an increase of the minimal inhibitory concentration (MIC) of the peptide up to 16-fold compared to the original strain (18). This increase is approximately four-fold stronger than what we observed in resistant strains we selected upon eight passages and that acquired point mutations in *envZ*, a gene encoding the osmosensor of a two-component system in *E. coli* (20). In order to investigate the cause of this strong increase in MIC, we performed whole genome sequencing on the P20 isolate to identify mutations, which may explain this high level of resistance. Three non-synonymous SNPs were identified in the genes *envZ*, *yrbL,* and *bamA*, and a deletion causing a premature stop codon was identified in the gene *skp* (Fig. 1*C*). One point mutation affects the periplasmic loop of EnvZ (EnvZ^E53G^). The second mutation is YrbL^E42G^, causing an amino acid change in a protein of unknown function, whose expression is regulated by Mg^2+^ through the PhoP/PhoQ two-component system (22). The third mutation is BamA^D498N^, and it removes a bacterial surface-located negative charge on an essential OMP part of the BAM complex. Finally, an 11-nucleotide deletion causes a frameshift and a premature stop codon in the *skp* gene, which encodes a periplasmic chaperone, part of the BAM machinery (23).

Having identified these mutations, we wanted to determine their order of acquisition during the selection process. We amplified the genes of interest by PCR and performed Sanger sequencing for all passages. EnvZ^E53G^ was the first mutation to appear (passage 5), followed by YrbL^E42G^ (passage 7). Deletion in *skp* (Skp^T40^^∗^) appeared at passage 12. The mutation in BamA was detected in passage 16 only (Fig. 1*C*). To exclude that undetected mutations or other factors may cause the observed resistance, we attempted to introduce individual mutations into *E. coli* using CRMAGE, a CRISPR-Cas9-based technique (24). We were able to construct strains with the single mutations YrbL^E42G^ and BamA^D498N^. However, despite several attempts, we could not generate *E. coli* with the EnvZ^E53G^ mutation. All clones obtained had a wild-type version of *envZ*, which might be explained by suboptimal targeting by the gRNA. We thus decided to use another mutation (EnvZ^D233N^) that we obtained using CRMAGE in a former study and that showed a similar level of resistance as the EnvZ^E53G^ mutation (20). We generated a strain with the single mutation EnvZ^D233N^ as well as a double mutant EnvZ^D233N^ BamA^D498N^. As a surrogate for SkpT40∗, we used the *Δskp* deletion mutant from the Keio collection (25) and introduced either EnvZ^D233N^ or BamA^D498N^ mutation in this deletion mutant. We analyzed the resistance of these mutants towards TAT-RasGAP_317-326_ and found that all four mutations together confer the highest level of resistance towards TAT-RasGAP_317-326_, whereas *Δskp* deletion mutants have resistance levels similar to that of the wild-type strain (Fig. 1*D*). BamA^D498N^ and YrbL^E42G^ single mutants show a limited increase of resistance in the wild-type background. BamA^D498N^ but not YrbL^E42G^ or *Δskp* mutations increased resistance in the EnvZ^D233N^ background (Fig. 1*D*).

### P20 strain shows growth defects and important changes in protein expression profile

To decipher the mechanisms by which the P20 strain shows a high level of resistance to TAT-RasGAP_317-326_, we decided to better characterize this mutant. We measured its growth rate and compared its morphology with the corresponding parental strain and individual mutants. Interestingly, strains with an EnvZ^D233N^ mutation had a significantly smaller size than strains with a wild-type version of EnvZ, as determined by cell area measurement (Fig. S1). In addition, the EnvZ^D233N^ mutation caused a slight growth defect. In contrast, the deletion of *skp* appeared to improve growth (Fig. S2), which might allow to partially compensate for the growth defect caused by the accumulation of mutations in the P20 strain.

**Figure 2:**
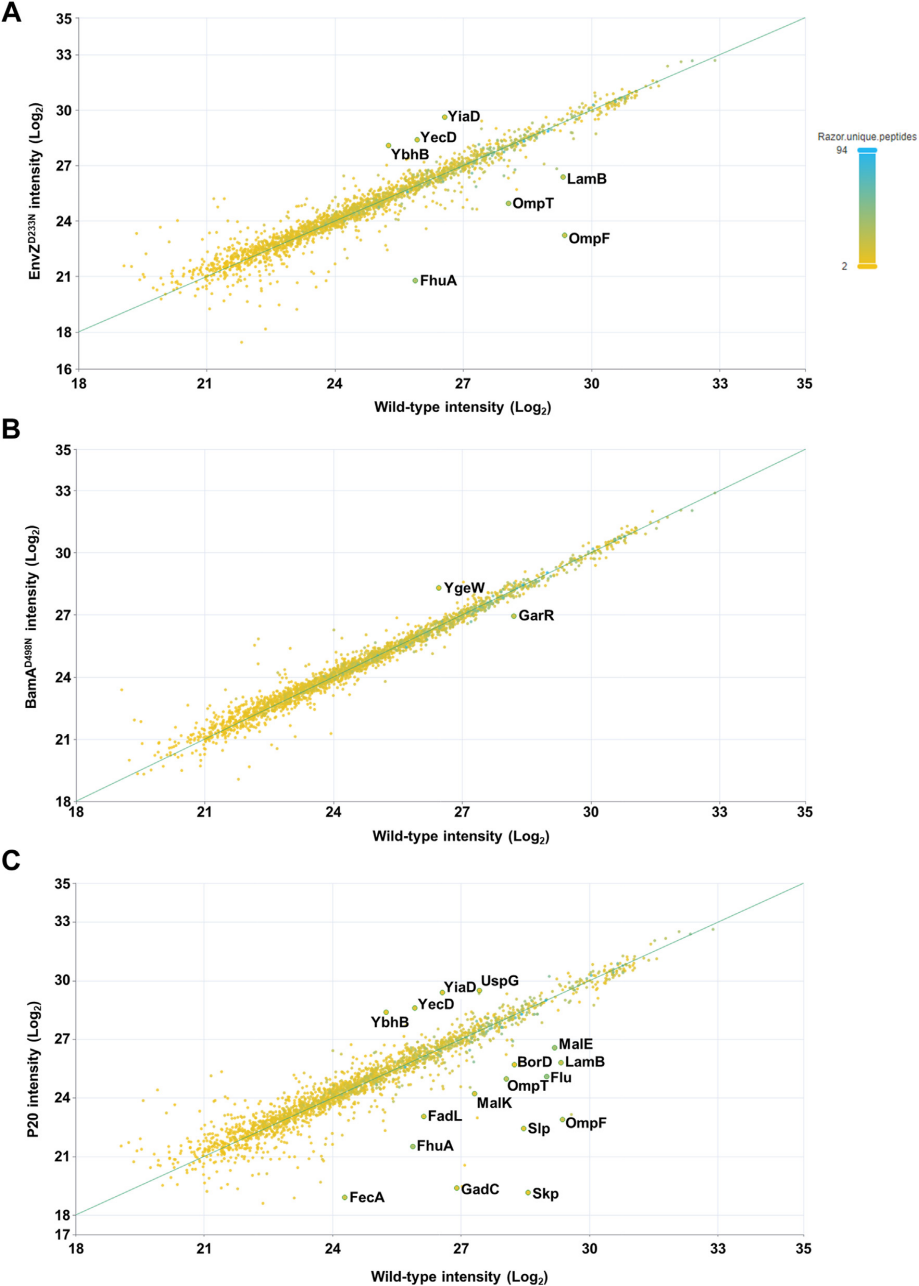
**EnvZ**^**D233N**^**and P20 mutants, but not BamA**^**D498N**^**, show decreased levels of some outer membrane proteins.** Equivalent quantities of bacteria as estimated by OD_600_ measurement were resuspended in Laemmli buffer and submitted to mass spectrometry. Levels of peptides of EnvZ^D233N^ mutant (*A*), BamA^D498N^ mutant (*B*) and P20 isolate (*C*) were compared to the wild-type. Proteins showing relevant differences of peptide levels compared to the wild-type are highlighted.

We then performed proteomic analyses to determine whether important changes in protein levels are induced by EnvZ^D233N^ and BamA^D498N^ mutations. We also analyzed the P20 strain and compared it with the single mutations (Fig. 2). Proteins that showed lower levels in the EnvZ^D233N^ mutant were mostly proteins involved in transport (OMPs, ABC transporters, Table S1), while enriched proteins were involved in stress response and antibiotic resistance (Table S2). The BamA^D498N^ mutation caused only very limited changes in protein levels (Tables S3 and S4). Interestingly, most of the protein level changes detected in EnvZ^D233N^ were also detected in the P20 strain. Additional changes detected only in the P20 strain include a strong decrease in peptides from Skp, which correlates with the premature stop codon caused by the deletion in *skp* gene found in this strain. Other important changes affected levels of transporters, acid-resistance proteins, and LPS biosynthesis enzymes (Tables S5 and S6). Taken together these results indicate that the accumulation of mutations causes changes in protein levels, indicative of a potential profound change in surface properties and stress status of the bacteria, possibly explaining the high-level resistance of P20 towards TAT-RasGAP_317-326_.

### Removal of negative charges at the surface of BamA increases resistance to TAT-RasGAP_317-326_

**Figure 3:**
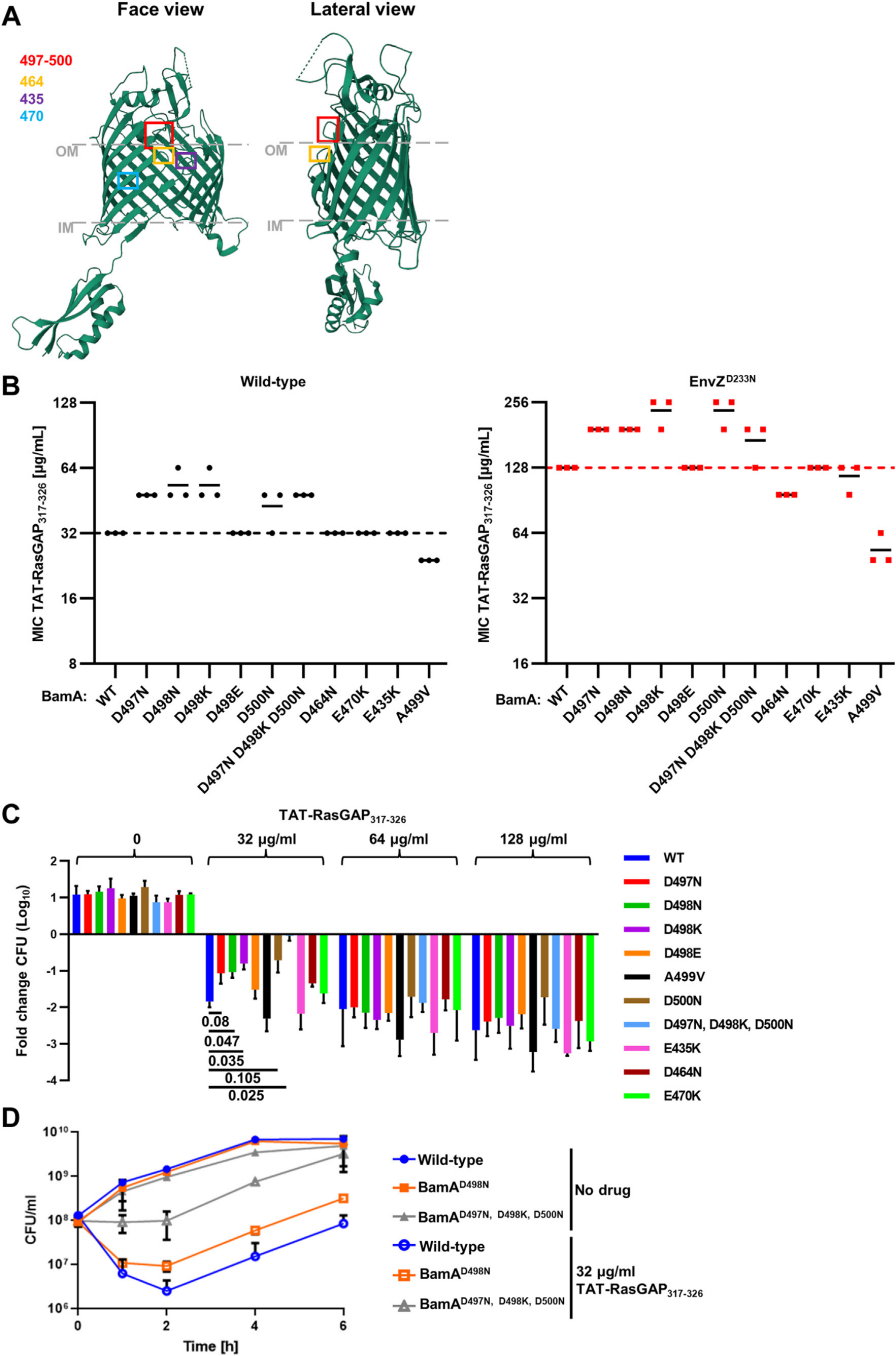
**Removal of negative charges at the surface of BamA increases resistance to TAT-RasGAP**_**317-326**_. *A*, mutation BamA^D498N^ is localized in an extracellular negatively charged loop of BamA. Negatively charged loop containing D498 residue, as well as other negatively charged residues (E435, E470, D464) mutated in this study were mapped on the 3D crystal structure of BamA (obtained from rcsb.org). *B*, removal of negative charges at the surface, but not on membrane-embedded domains causes an increased resistance to TAT-RasGAP_317-326_. Indicated point mutants were designed by CRMAGE and the MICs of TAT-RasGAP_317-326_ towards them was measured in triplicate. The dashed line corresponds to the average MIC of the wild-type (left) or the EnvZ^D233N^ mutant (right). Continuous lines represent the average MIC for the three replicates. *C*, removal of negative charges at the surface of BamA increases survival of *E. coli* to an intermediate concentration of TAT-RasGAP_317-326_. Overnight cultures of the indicated strains were diluted to 0.1 OD_600_ and grown for 1 hour before addition of different concentrations of the peptide. CFU were measured at time of addition of the peptide and after 2h of incubation with the peptide. Fold change CFU(2h)/CFU(0h) is displayed. Experiment was performed in triplicate and error bars represent standard deviations. *p* values shown were calculated using a *t* test. *D*, survival to TAT-RasGAP_317-326_ increases proportionally with the number of negative charges removed at the surface of BamA. The indicated *E. coli* strains were treated as for (*C*), but incubated with or without the peptide for 0h, 1h, 2h, 4h and 6h. CFU were measured in triplicates at these time points and error bars represent standard deviation.

The BamA^D498N^ mutation did not cause strong changes in the proteomic profile of *E. coli* (Fig. 2*B*). We thus wanted to determine the role of this mutation in the resistance to TAT-RasGAP_317-326_. BamA is a well-described essential protein, and its crystal structure is available (26). Based on this knowledge, we could map the BamA^D498N^ mutation to a highly negatively charged loop (composed of amino acids 495–505, with a net charge of −4) in a portion of the protein exposed at the surface of the bacterium (Fig. 3*A*). Because the D498N mutation constitutes a change from a negative to a neutral amino acid, we hypothesized that this change might impact the resistance of *E. coli* to TAT-RasGAP_317-326_ by reducing the interaction between this region and the positively charged TAT-RasGAP_317-326_. Using CRMAGE, we designed mutants in this loop, either removing one negative charge (BamA^D497N^, BamA^D500N^), replacing a negative charge with a positive charge (BamA^D498K^), or replacing three negative charges with one positive charge (BamA^D497N, D498K, D500N^). In addition, we replaced the negatively charged amino acid in position 498 with another negatively charged one (D498E) and finally removed or exchanged negative charges in other positions (D464N, E470K, E435K). We observed a limited, but clear and reproducible increase in the MIC of TAT-RasGAP_317-326_ linked to the removal of negative charges at the surface of BamA (Fig. 3*B*). However, the triple mutant BamA^D497N, D498K, D500N^ with a +4 charge change in the loop region did not show a higher MIC than the single mutants. These results could be replicated in an EnvZ^D233N^ background, indicating that there is an additive effect of BamA and EnvZ point mutations (Fig. 3*B*). Point mutations of BamA we investigated here did not influence bacterial fitness, since we did not observe any growth defects (Fig. S3) and phenotypic changes (Fig. S4) of these mutants compared to the wild-type strain. Moreover, protein levels of BamA and OmpC (an example of a BamA substrate) were comparable in all mutants, suggesting that the resistance to TAT-RasGAP_317-326_ is not associated with changes in protein levels and activity of BamA (Fig. S5).

Detailed examination of bacterial viability of the BamA point mutants in the presence of TAT-RasGAP_317-326_ through quantification of colony-forming units (CFU) was consistent with data from the MIC measurements. We found that only mutants affecting the charge of the Q495-T505 loop of BamA showed an effect on bacterial viability in the presence of TAT-RasGAP_317-326_ (Fig. 3*C*). This effect was limited to an intermediate concentration of TAT-RasGAP_317-326_ (32 μg/ml) and was especially striking for the triple mutant BamA^D497N, D498K, D500N^, which showed no change in viability at this concentration. This effect was confirmed by determining the survival rate of the triple mutant and comparing it with that of a wild-type strain and the BamA^D498N^ mutant at different time points (2h, 4h, 6h) (Fig. 3*D*). Interestingly, the effects of BamA mutations on TAT-RasGAP_317-326_ MIC levels and bacterial viability in the presence of this peptide required the presence of the *envZ* gene. Indeed, the insertion of BamA point mutation in an *envZ* deletion mutant did not alter the sensitivity of this strain to TAT-RasGAP_317-326_ (Fig. S6).

### BamA point mutants remain susceptible to other antimicrobial agents

**Figure 4:**
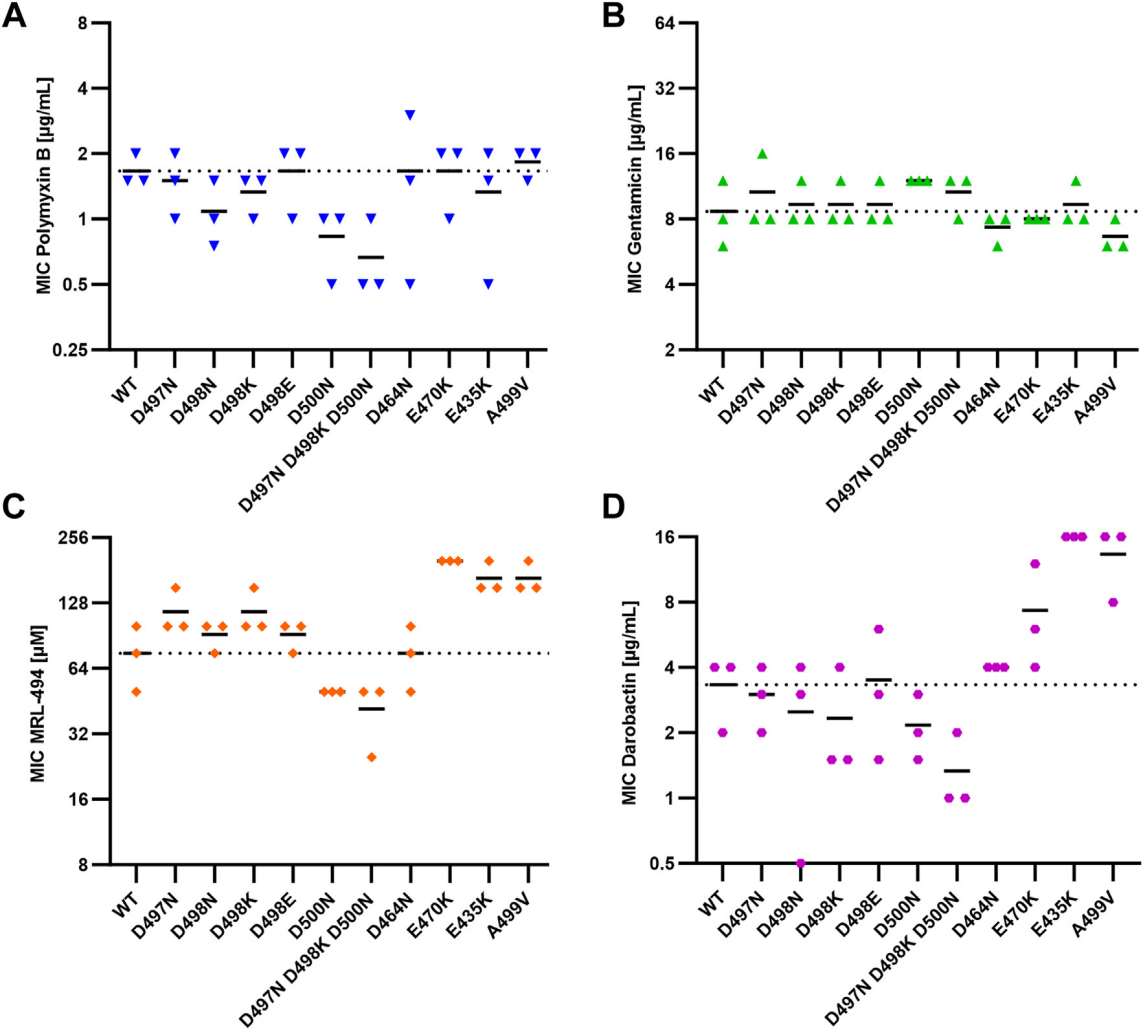
**BamA point mutations increasing resistance to TAT-RasGAP**_**317-326**_**do not cause broad-range resistance to AMPs and classical antibiotics but can influence the efficiency of BamA inhibitors.** MICs of the AMP polymyxin B (*A*), the antibiotic gentamicin (*B*), the BAM complex inhibitors MRL-494 (*C*), and darobactin (*D*) on the indicated strains were measured in triplicate. Dashed lines correspond to the average MIC of the wild-type. Continuous lines represent the average MIC for the three replicates.

Next, we measured the susceptibility of BamA point mutants to the AMP polymyxin B and the antibiotic gentamicin (Fig. 4, *A* and *B*). No increased resistance could be detected in these mutants, excluding a broad-range resistance phenotype that could have been caused by changes in the permeability of the outer membrane. In contrast, the BamA point mutations showed diverse effects on resistance towards known BamA inhibitors. Some BamA mutations causing resistance to TAT-RasGAP_317-326_ (D497N, D498K) were associated with increased resistance to MRL-494 but not to darobactin. Additionally, mutations that previously were described to cause resistance to MRL-494 and darobactin (E470K, E435K) (10, 27) did not alter the resistance level of *E. coli* to TAT-RasGAP_317-326_ (Figs. 3*B* and 4, *C* and *D*). Based on these data, we found no clear correlation between sensitivity to TAT-RasGAP_317-326_ and to other antimicrobial agents in the BamA point mutants we tested.

### Resistance caused by BamA mutations requires the presence of other members of the BAM complex

**Figure 5:**
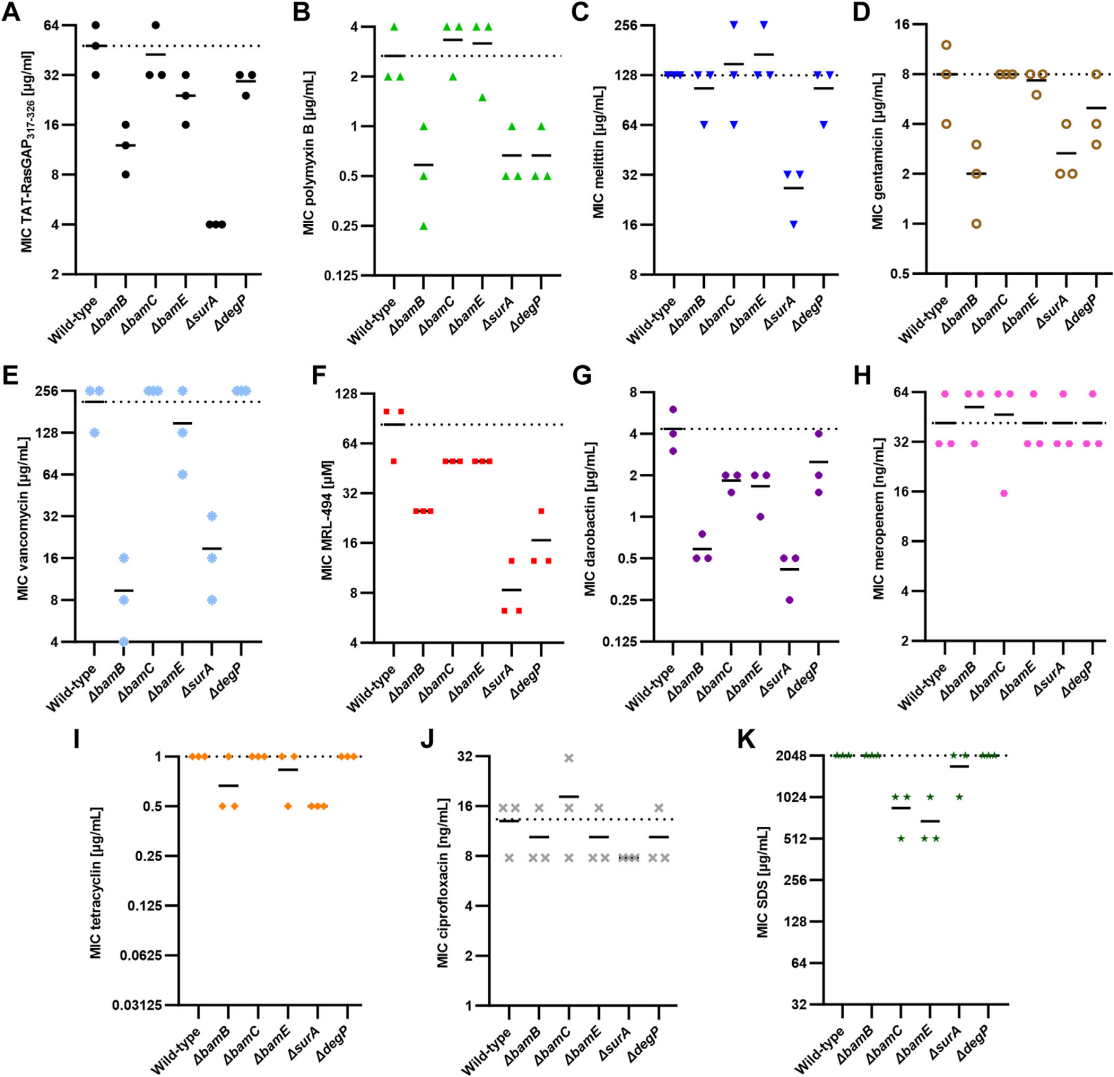
**Deletion of non-essential components of the BAM complex does not result in resistance to TAT-RasGAP_317-326_ but can increase sensitivity to diverse antimicrobial agents.** MICs of TAT-RasGAP_317-326_ (*A*), polymyxin B (*B*), melittin (*C*), gentamicin (*D*), vancomycin (*E*), MRL-494 (*F*), darobactin (*G*), meropenem (*H*), tetracycline (*I*), ciprofloxacin (*J*), and SDS (*K*) on the indicated deletion mutants from the Keio collection. MICs were measured in duplicate. Dashed lines correspond to the average MIC of the wild-type. Continuous lines represent the average MIC for the three replicates.

We then tested the influence of deleting the non-essential components of the BAM complex on the sensitivity of *E. coli* to TAT-RasGAP_317-326_. Deletion mutants were taken from the Keio collection (25) and verified by PCR. Deletions of *bamC*, *bamE,* and *degP* did not cause strong changes in sensitivity levels to TAT-RasGAP_317-326_, but deletions of *bamB* and *surA* caused an increased sensitivity to the peptide (Fig. 5*A*). Interestingly, similar effects were observed regarding susceptibility to other AMPs such as polymyxin B and melittin (Fig. 5, *B* and *C*), the positively charged antibiotic gentamicin (Fig. 5*D*), the high molecular weight antibiotic vancomycin (Fig. 5*E*) and BamA inhibitors MRL-494 and darobactin (Fig. 5, *F* and *G*). In contrast, no changes in sensitivity levels to other antibiotics and to SDS were observed (Fig. 5, *H*–*K*).

**Figure 6:**
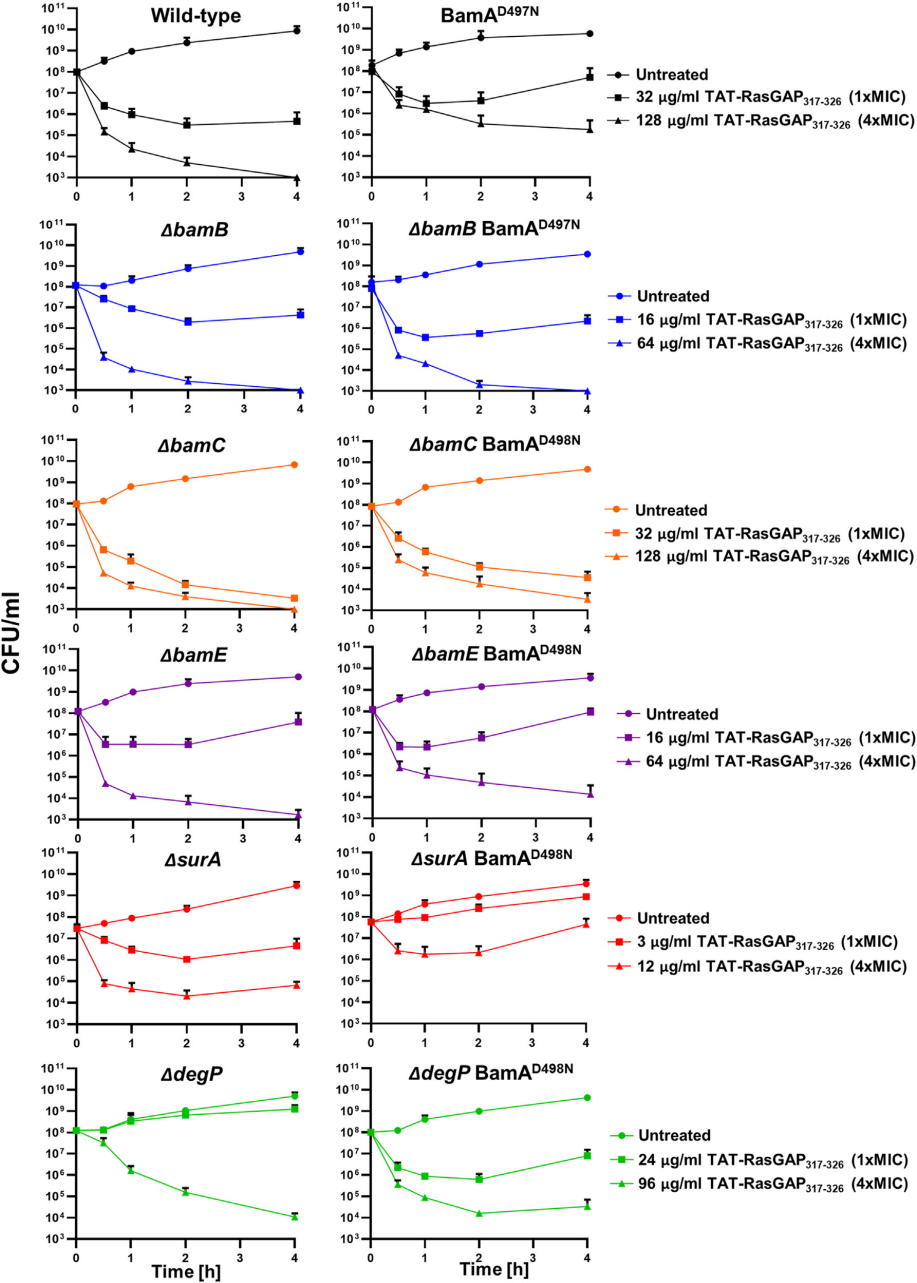
**Some non-essential components of the BAM complex are required for resistance conferred by BamA mutations.** BamA^D498N^ mutation was inserted in deletion mutants of BAM complex components. In the *ΔbamB* mutant, a BamA^D498N^ mutation could not be obtained and had to be replaced by a BamA^D497N^ mutation. CFU were measured on the deletion mutants containing or not the BamA point mutation at the indicated times after treatment with concentrations of TAT-RasGAP_317-326_ corresponding to 1x or 4x the MIC. Experiments were done in triplicates and error bars represent standard deviations.

We then wondered whether non-essential subunits of the Bam complex were required for the resistance induced by point mutations in *bamA*. We used CRMAGE to create BamA point mutations in *E. coli* strains possessing a deletion of the various BAM complex proteins. Mutants were treated with one or 4 times the MIC of TAT-RasGAP_317-326_ toward the corresponding deletion mutant, as measured in Figure 5*A*. Influence of deleting BAM complex subunits on survival of the BamA point mutant was then quantified by CFU measurement (Fig. 6). Interestingly, we did not observe an increased survival of bacteria having a BamA point mutation in the absence of BamB, BamC or DegP, indicating that these subunits are required for resistance in the BamA point mutant. Deletion of BAM complex components might influence resistance in the BamA point mutant *via* destabilization of the BAM complex. Such a destabilization would cause a decrease in BamA levels as well as in the levels of other OMPs. To assess this, we measured protein levels of BamA and OmpC in the different deletion mutants and observed no clear correlation between protein levels and the influence of the gene deletion on resistance towards TAT-RasGAP_317-326_ (Fig. S7). This result indicates that the deletion of BAM complex components does not abolish resistance to TAT-RasGAP_317-326_ through changes in protein level or protein activity.

### *In silico* modeling predicts an interaction between TAT-RasGAP_317-326_ and the 495 to 505 loop of BamA

**Figure 7:**
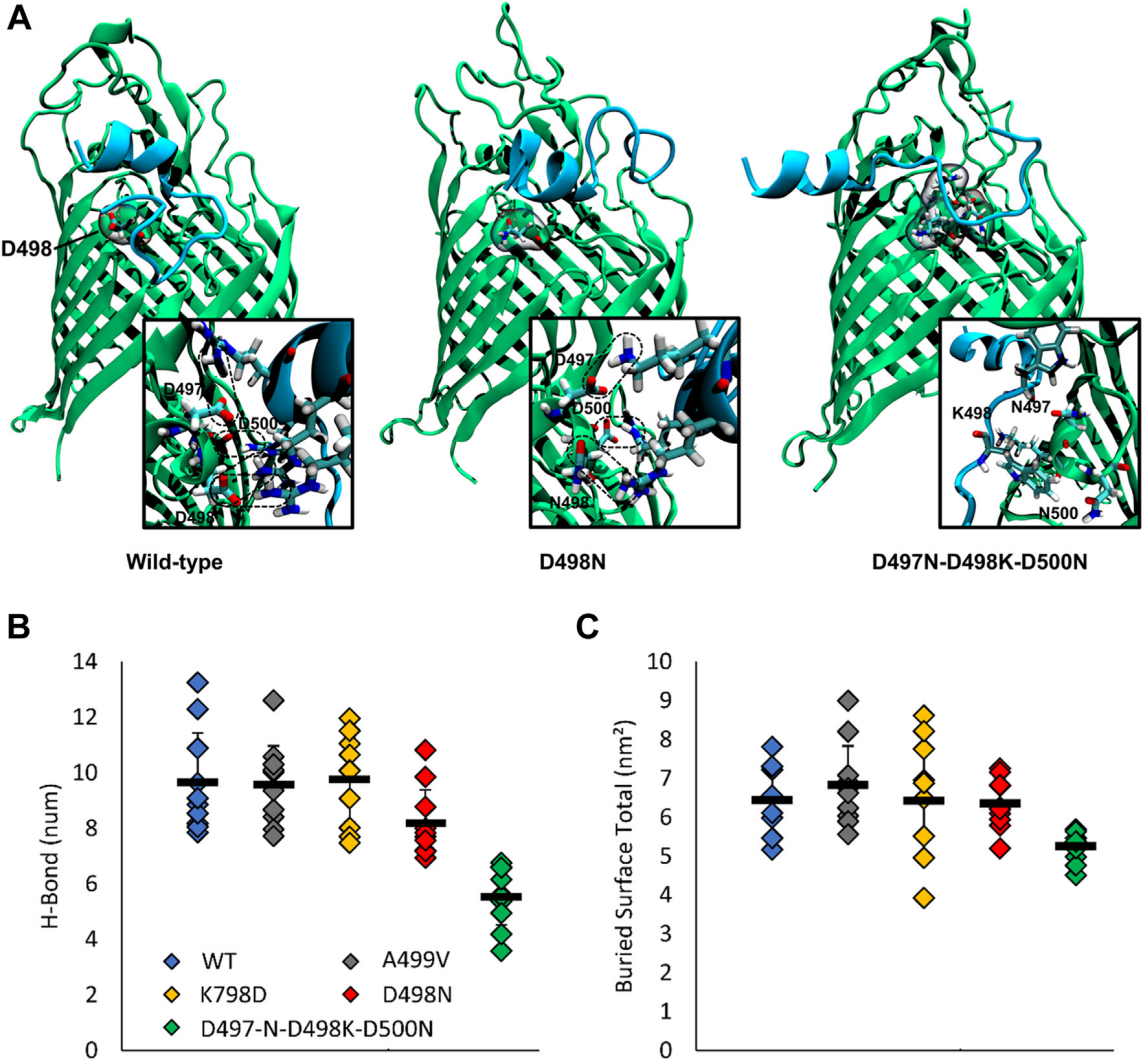
***In silico* modelling predicts possible interaction between TAT-RasGAP**_**317-326**_**and the 495 to 505 amino acids loop of BamA**. *A*, qualitative representation of the best interacting poses of TAT-RasGAP_317-326_ and the indicated versions of BamA after molecular dynamics simulations. The D498 amino acid of the wild-type, as well as the D498 N and D497N-D498K-D500 N mutations, are highlighted on the respective BamA structures. In addition, a zoom-in image of each case is shown to enhance the clarity of the interactions between the peptide and BamA. A qualitative representation of the possible H-bond interactions between the BamA’s D497, D498, and D500 amino acids, or the respective mutations and TAT-RasGAP_317-326_ is shown using dashed boxes. *B*, H-bonds analysis between TAT-RasGAP_317-326_ and BamA and (*C*) Buried Surface analysis between TAT-RasGAP_317-326_ and BamA. The diamond marks represent the average values of H-Bonds and Buried Surface of the three replica averages (computed in the last 20 ns of simulated time). In total, there are 10 diamond marks for each system (WT, A499V, K798D, D498N, D497N-D498K-D500N), one for each starting docking pose. Finally, the black dashes represent the average values of all the diamond marks for each system.

Since the removal of negative charges at the surface of BamA affects the efficiency of TAT-RasGAP_317-326_, we hypothesized that the positively charged TAT-RasGAP_317-326_ directly interacts with BamA and that this interaction may be important for its activity. To address this, we used a combined *in silico* modeling approach to simulate the interaction between TAT-RasGAP_317-326_ and BamA embedded in the outer membrane. This was performed using a molecular docking procedure and a subsequent classical molecular dynamics protocol, in which all the docking poses were simulated. The last 20 ns of the 200 ns production run of each simulation were considered for the analyses (see Methods section). Interestingly, a favorable interaction between TAT-RasGAP_317-326_ and the 495 to 505 loop was predicted (Fig. 7*A*). The same simulation was then performed using BamA^D498N^ and BamA^D497N, D498K, D500N^ mutants (Fig. 7*A*). Both the number of hydrogen bonds predicted between the peptide and the protein (Fig. 7*B*) as well as the predicted buried surface (Fig. 7*C*) were lower for the mutants than the wild-type, indicating a lower interaction tendency between the peptide and the protein. It is worth noting that the number of hydrogen bonds was lower in BamA^D498N^ and BamA^D497N, D498K, D500N^ mutants in comparison to the wild-type, remarking the pivotal role played by electrostatic interactions (Fig. 7*B*). In a further step, we predicted point mutations that would induce a higher binding affinity between BamA and TAT-RasGAP_317-326_. Two mutations were predicted to have this effect - A499V and K798D (Fig. 7, *B* and *C*). We tried to produce these mutants and could obtain only the A499V mutant. Despite the encouraging molecular modeling prediction, we could not obtain a mutant with BamA^K798D^, indicating that this mutation might affect a residue that is essential for BamA activity. A499V mutation caused increased sensitivity to TAT-RasGAP_317-326_ (Fig. 3*B*) consistent with the *in silico* prediction of increased interaction capability in the context of this mutation (Fig. 7*C*). The A499V mutation enhanced the capacity of TAT-RasGAP_317-326_ to extend its interaction surface compared to the wild-type, while maintaining comparable hydrogen bond interactions (Fig. 7, *B* and *C*). This suggests that the A499V mutation stabilizes the hydrophobic interactions within the 495 to 505 loop, thereby fostering an increased buried surface area between the peptide and the protein.

### TAT-RasGAP_317-326_ effect on BamA activity is not sufficient to explain its antibacterial activity

**Figure 8:**
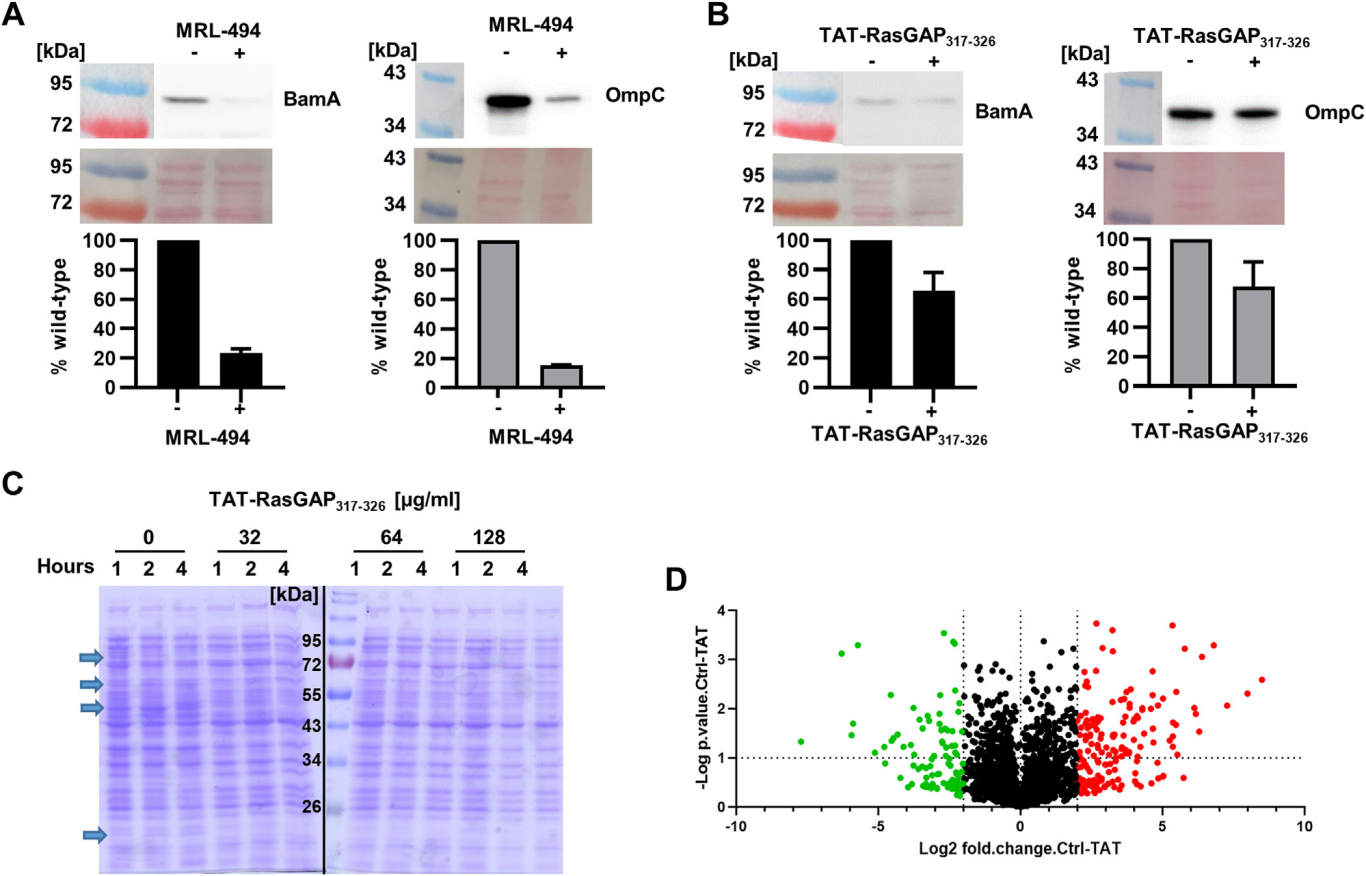
**BamA activity is only partially reduced in presence of TAT-RasGAP**_**317-326**_. *A*, the known BamA inhibitor MRL-494 strongly reduces BamA and OmpC protein levels. Exponential phase wild-type *E. coli* were treated or not with 100 μM MRL-494 for 1 hour. OD_600_ was then measured, and equivalent quantities of bacteria were harvested and resuspended in Laemmli buffer. A Western-blot was then performed with antibodies targeting BamA or OmpC, respectively. Analysis was performed using ImageJ and intensity of the bands was compared to the untreated control. Experiments were performed in duplicate; one representative result is shown and average of the two quantifications are provided. *B*, TAT-RasGAP_317-326_ only partially reduces BamA and OmpC protein levels. Bacteria were treated or not with 32 μg/ml TAT-RasGAP_317-326_. Experiment was performed and analysed as for (A). *C*, protein profile is affected by TAT-RasGAP_317-326_ treatment. Exponential phase bacteria were treated with the indicated concentrations of TAT-RasGAP_317-326_ for the indicated periods of time. Equivalent quantities of bacteria (as determined by OD_600_ measurement) were resuspended in Laemmli buffer and analysed by SDS-PAGE and Coomassie staining. *Arrows* indicate bands, which levels visibly decrease upon peptide treatment. *Black line* indicates separation between two different gels. *D*, TAT-RasGAP_317-326_ treatment has a profound effect on protein composition of *E. coli*. Bacteria treated for 1 hour with or without 32 μg/ml TAT-RasGAP_317-326_ were submitted to mass spectrometry analysis. Experiment was performed in duplicates and Fold-change of protein levels were determined. Proteins with Log_2_ of fold change higher than two or lower than −2 are highlighted in *red and green*, respectively.

Since a direct binding of TAT-RasGAP_317-326_ with BamA was predicted, we wondered whether this interaction may affect the activity of BamA. It has been shown by others that, upon inhibition of BamA, OMPs, such as OmpC and BamA itself, are not inserted properly in the OM and are degraded (27). Consistently, treatment of *E. coli* with the BamA inhibitor MRL-494 caused a strong decrease of both BamA and OmpC protein levels (approx. 80% decrease, Fig. 8*A*). In contrast, treatment with TAT-RasGAP_317-326_ induced only a limited decrease of BamA and OmpC protein levels (approx. 30% decrease, Fig. 8*B*). To confirm this result, we performed the same experiment with increasing concentrations of TAT-RasGAP_317-326_ and with different incubation times. Increasing concentrations of TAT-RasGAP_317-326_ caused a visible reduction of BamA levels (Fig. S8*A*). However, OmpC levels were only partially affected by this treatment (approx. 50% decrease, Fig. S8*B*). In a further step, we compared the morphology of *E. coli* treated or not with TAT-RasGAP_317-326_, MRL-494 and darobactin. We observed very different phenotypes upon treatments with TAT-RasGAP_317-326_ and with BamA inhibitors, further indicating that TAT-RasGAP_317-326_ may not be a BamA inhibitor (Fig. S9). We still could not exclude that TAT-RasGAP_317-326_ partially affects BAM complex activity, since we tested only protein levels of BamA and OmpC. To determine whether levels of other proteins were affected by TAT-RasGAP_317-326_ treatment, we performed an SDS-PAGE followed by Coomassie staining. We could clearly observe different band levels between untreated and treated samples (Fig. 8*C*). We thus performed proteomic analyses of the same samples (Fig. 8*D*). Interestingly, we did not observe a strong general decrease in protein levels of components of the BAM complex and of a selection of OMPs (Table 1). Nevertheless, the slight decrease of OmpC level that we observed by Western blotting (Fig. 8*B*) was confirmed in our proteomic data (Table 1). Looking in more detail at these data, we observed a general decrease in the levels of proteins involved in catabolic pathways and regulated by CRP (Tables S7 and S8 and Dataset 2). These results are in favor of a model in which the BAM complex participates in the binding and translocation of TAT-RasGAP_317-326_ through the OM but seem to exclude a direct role of TAT-RasGAP_317-326_ on the activity of the Bam complex. Further studies are now needed to decipher the exact mode of action of TAT-RasGAP_317-326_ and its possible link with CRP-regulated genes.

**Table 1:** Influence of TAT-RasGAP_317-326_ on OMPs protein levels

Protein IDs	Protein names	Gene names	Mol. Weight [kDa]	Log2 fold change (Ctrl-TAT)	Untreated replicate A	TAT-RasGAP replicate A	Untreated replicate B	TAT-RasGAP replicate B
P66948	Beta-barrel assembly-enhancing protease	*bepA*	53.91	3.02	13	8	12	8
P0A937	Outer membrane protein assembly factor BamE	*bamE*	12.30	1.48	2	5	5	6
P02931	Outer membrane protein F	*ompF*	39.33	1.19	38	28	47	32
P06996	Outer membrane protein C	*ompC*	40.37	0.83	203	147	260	190
P31554	LPS-assembly protein LptD	*lptD*	89.67	0.62	40	36	44	39
P02930	Outer membrane protein TolC	*tolC*	53.74	0.56	34	41	39	38
P05825	Ferrienterobactin receptor	*fepA*	82.11	0.46	1	8	0	6
P06129	Vitamin B12 transporter BtuB	*btuB*	68.41	0.43	30	29	37	31
P0A915	Outer membrane protein W	*ompW*	22.93	0.26	17	14	18	16
P0A903	Outer membrane protein assembly factor BamC	*bamC*	36.84	0.23	16	21	24	24
P0A910	Outer membrane protein A	*ompA*	37.20	0.13	78	58	78	66
P0ABZ6	Chaperone SurA	*surA*	47.28	0.10	29	26	34	28
P0ADE4	Translocation and assembly module TamA	*tamA*	64.80	0.10	5	4	5	7
P0AEU7	Chaperone protein Skp	*skp*	17.69	0.02	10	16	17	12
P09169	Protease 7	*ompT*	35.56	−0.03	25	32	35	35
P0A940	Outer membrane protein assembly factor BamA	*bamA*	90.55	−0.07	54	54	60	57
P31600	Bacteriophage N4 adsorption protein A	*nfrA*	111.31	−0.11	3	2	3	3
P0AC02	Outer membrane protein assembly factor BamD	*bamD*	27.83	−0.27	12	16	16	13
P0C0V0	Periplasmic serine endoprotease DegP	*degP*	49.35	−0.39	37	47	37	50
P10384	Long-chain fatty acid transport protein	*fadL*	48.54	−0.42	22	20	23	25
P76115	Probable TonB-dependent receptor YncD	*yncD*	77.26	−0.49	3	6	6	10
P77774	Outer membrane protein assembly factor BamB	*bamB*	41.89	−0.63	19	21	25	24
P76471	Uncharacterized protein YfaZ	*yfaZ*	18.61	−0.84	3	2	4	3
P0A917	Outer membrane protein X	*ompX*	18.60	−0.97	35	55	42	55
P13036	Fe(3+) dicitrate transport protein FecA	*fecA*	85.32	−1.52	5	14	5	24
P06971	Ferrichrome-iron receptor	*fhuA*	82.18	−2.29	21	51	20	45
P75780	Catecholate siderophore receptor Fiu	*fiu*	81.96	−2.84	1	9	2	10
P21420	Putative outer membrane porin protein NmpC	*nmpC*	40.30	−3.04	4	10	5	11
P02929	Protein TonB	*tonB*	26.09	−3.17	1	1	1	1
P17315	Colicin I receptor	*cirA*	73.90	−4.79	1	10	0	9

Proteomic analysis of changes of OMP protein levels between *E. coli* treated or not with TAT-RasGAP_317-326_. Data were extracted from Dataset 2.

### The combination of TAT-RasGAP_317-326_ and MRL-494 is synergistic

**Figure 9:**
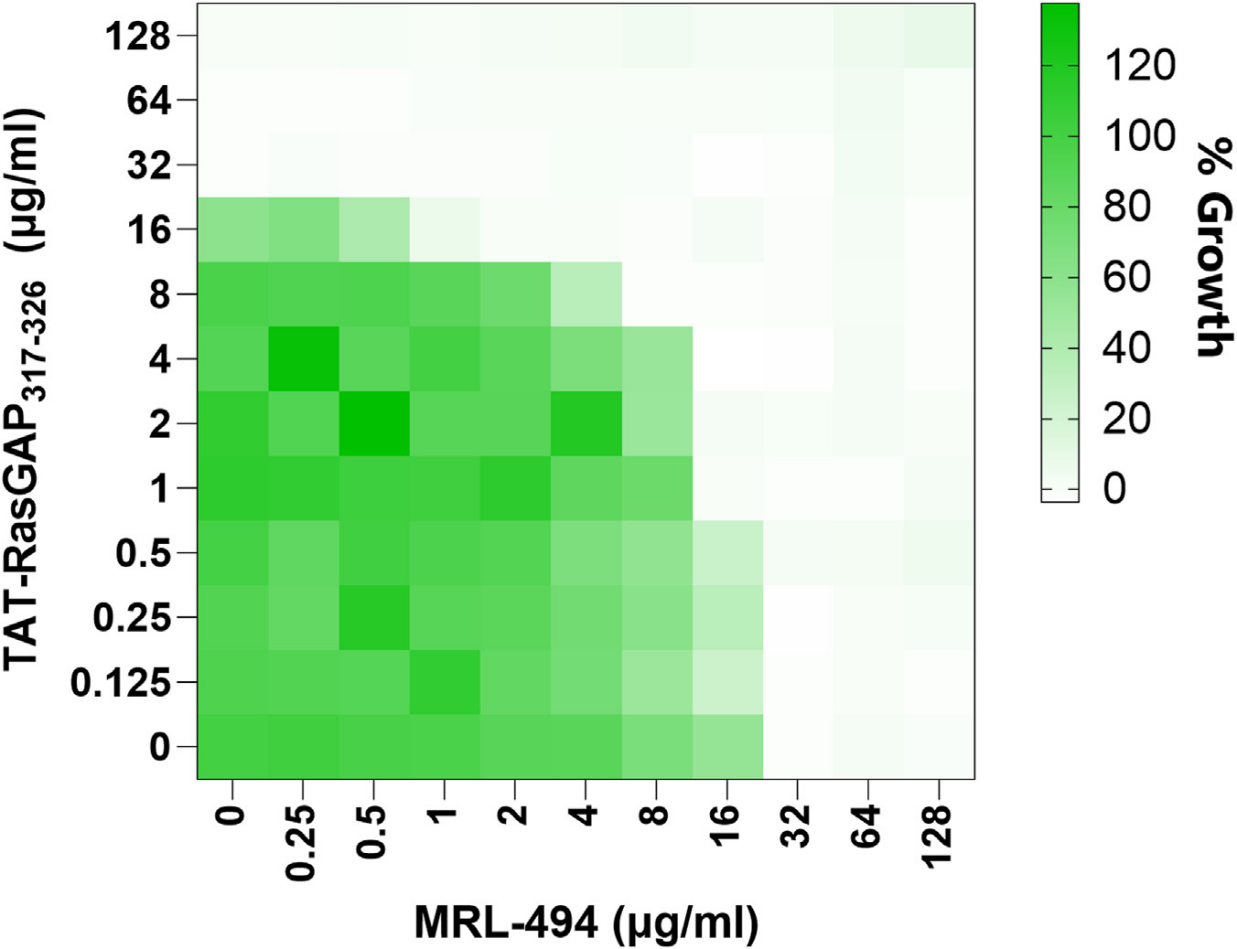
**The combination between TAT-RasGAP**_**317-326**_**and the BAM complex inhibitor MRL-494 is synergistic.** Checkerboard assay was performed using *E. coli* wild-type strain with the indicated concentrations of antimicrobial agents. Growth was monitored through OD_600_ measurement and represented as a percentage of the untreated control. Synergism was determined with a FICI of 0.5, as detailed in the methods section. The experiment was performed in triplicate; a representative experiment is shown.

Since we observed that some deletion mutants of the BAM complex were hypersensitive to TAT-RasGAP_317-326_, we tested a combination of BAM complex inhibitor and TAT-RasGAP_317-326_ using a checkerboard assay (Fig. 9). We observed synergism (FICI=0.5) between these two compounds, indicating that such combinations may be a promising strategy to potentiate the activity of antimicrobial peptides and avoid the development of resistance.

## Discussion

The antibacterial mechanism of action (MOA) of TAT-RasGAP_317-326_ remains elusive, despite several attempts to decipher it (18, 20, 28). Data acquired up to now indicate that MOA of this peptide may be intracellular since bacterial death occurs only approximately 1 hour after treatment (18). In addition, recent investigations showed that TAT-RasGAP_317-326_ kills cancer cells by acting on the inner leaflet of the plasma membrane (17). In this study, we investigate resistance emergence toward TAT-RasGAP_317-326_, which could also help to get insights into the MOA of this peptide. To address these questions, we performed *in vitro* resistance selection (18) where we isolated a resistant strain carrying three non-synonymous point mutations in the genes *envZ*, *yrbL,* and *bamA* and a deletion causing a premature stop codon in the *skp* gene.

We previously described mutations in *envZ*, coding for the osmosensor of a two-component system, that cause resistance to TAT-RasGAP_317-326_ (20). Interestingly, the mutation detected in this study (E53G) is localized in the periplasmic loop of EnvZ, while mutations found previously were all localized in the cytoplasmic dimerization domain (D233N, V241G, P248A, D273E). The mutation found in YrbL (E42G) was also reminiscent of a mutation found in our earlier study (V149L). YrbL function is not known, but its expression is regulated by Mg^2+^ through the PhoP/PhoQ two-component system (22). Skp is a chaperone involved in outer membrane protein biogenesis (23, 29). Its deletion increases the growth rate and might thus improve the growth rate of the P20 mutant, compensating for the fitness cost due to the accumulation of mutations.

The fourth mutation, BamA^D498N^, was not detected in earlier studies. Acquisition of a point mutation in the essential gene *bamA*, encoding an OMP involved in proper folding and localization of OMPs, led us to hypothesize that TAT-RasGAP_317-326_ might have a direct effect on this protein. However, results presented in this study indicate that BamA activity is only partially affected by TAT-RasGAP_317-326_. This effect is not sufficient to explain the antimicrobial activity of this peptide. BamA may thus not be the direct target of the peptide, but an intermediary to facilitate peptide activity.

The effect of BamA point mutations on bacterial survival rate in the presence of TAT-RasGAP_317-326_ was observed only with intermediate concentrations of the peptide (Fig. 3*C*). This might indicate that, at high concentrations, the peptide bypasses the need for interaction with BamA, either because its high concentration compensates for its lower affinity or because a high concentration of TAT-RasGAP_317-326_ causes additional antimicrobial effects. A similar phenomenon was shown for other AMPs, which can have intracellular targets at low concentrations, but have a membrane lytic activity at high concentrations (30).

The observation that resistance requires the presence of other components of the BAM complex is puzzling. This seems to indicate that a fully functional BAM complex is necessary for resistance to TAT-RasGAP_317-326_, which would be in line with the effect of the peptide on the activity of BamA. However, it is also possible that the conformation of BamA, and thus its binding affinity to TAT-RasGAP_317-326_, is influenced by the presence of other components of the BAM complex. However, this is highly speculative and would need to be investigated in the future.

We showed that the optimal efficiency of TAT-RasGAP_317-326_ towards *E. coli* depends on its affinity to a negatively charged loop at the surface of BamA. This indicates that BamA is not only an essential insertase and a potential drug target but may also work as a receptor for some AMPs, such as TAT-RasGAP_317-326_. Transient interaction of this peptide with BamA may help the peptide to cross the OM, either directly through the membrane or through BamA itself, during the insertion of OMPs in the OM. We could use this knowledge in the future to modify TAT-RasGAP_317-326_ to increase its affinity for bacteria and thus improve its efficacy. Interestingly, combining TAT-RasGAP_317-326_ with BAM inhibitors may be a promising strategy to potentiate its activity, similar to what we already described for combinations between TAT-RasGAP_317-326_ and antibiotics (21). Whether such combinations can avoid the emergence of resistance needs to be investigated in the future.

## Experimental procedures

### Strains and cultural conditions

*E. coli* strains used in this study are listed in Table S9. Strains were grown in LB (1% (w/v) tryptone, 0.5% yeast extract, 1% NaCl) at 37°C with constant shaking (220 rpm) or stationary on Petri dish containing LB supplemented with 1.5% agar (LBA) at 37°C. Strains were stored at −80°C upon addition of 16% glycerol final concentration to overnight (ON) liquid LB cultures.

### Peptides and other antimicrobial agents

TAT-RasGAP_317-326_ peptide was synthesized as a retro-inverso version (amino acid sequence DTRLNTVWMWGGRRRQRRKKRG, reversed sequence and chirality compared to the original peptide) by SBS Genetech. Polymyxin B, gentamicin, and meropenem were provided by Merck (Sigma-Aldrich). Vancomycin, tetracycline, and ciprofloxacin were purchased from Applichem. MRL-494 was provided by Medchemexpress and melittin by Enzo Life Sciences. Darobactin was kindly provided by the laboratory of Kim Lewis (Northeastern University).

### Selection of resistant strains

Selection of resistant strains was performed by diluting 100x an overnight culture of MG1655 *E. coli* in LB with 0.5x MIC TAT-RasGAP_317-326_ along with a no peptide control culture. The *E. coli* cultures were grown overnight and, the following morning, diluted again 100x in the presence of the same or an increased concentration of TAT-RasGAP_317-326_. Once growth was detected in the bacterial culture exposed to increased concentration of the peptide, the culture was propagated again, and the concentration of peptide was further increased. This cycle was repeated for a total of 20 passages and whole genome sequencing was performed on the final passage to identify acquired genetic mutations in comparison to the original MG1655 *E. coli* strain.

### MIC measurements

An overnight culture of the tested strain in LB at 37 °C was diluted to 0.1 OD_600_ and grown for 1 hour. The culture was then diluted 20 times in fresh LB and 10 μl of the diluted culture were added to wells of a 96-well plate in which two-fold dilutions of a given antimicrobial agent in 100 μl LB were prepared. The plate was then incubated for 16 h statically at 37 °C in a humidified atmosphere and growth was measured by OD_590_ measurement using a FLUOstar Omega microplate reader (BMG Labtech). Growth control in the absence of antimicrobial agents and sterility control were performed for each experiment. Minimal inhibitory concentration (MIC) was defined as the minimal concentration of antimicrobial agent required to inhibit growth by at least 90%.

### Whole genome sequencing

Genomic DNA was extracted using a Wizard genomic purification kit (Promega) and quantified using a Qubit system (Thermo Fisher). Libraries were created with a Nextera XT Kit (Illumina) and their quality was controlled using a Fragment Analyzer AATI (Agilent). Sequencing was then performed on a MiSeq system (Illumina) using MiSeq Reagent Kits v2. The obtained reads were assembled using spades v.3.11.1 (31) and mapped on a corresponding reference genome with bwa v.0.7.17 (32). Mutations were identified by variant calling, using gatk 4.0.2.0 (33). A threshold was used to validate mutations, by considering the ones that were supported by at least 75% of reads, with a minimal sequencing depth of 10. This was then manually confirmed using JBrowse (34).

### PCR and Sanger sequencing

Sequencing of individual genes of interest was performed by PCR amplification of the gene and subsequent Sanger sequencing. PCR was performed with control primers listed in Table S1. GoTaq G2 Hot Start Master Mix (Promega) was used with 0.2 μM of each primer (for *yrbL* and *bamA*) or 1 μM of each primer (for *envZ* amplification). Using sterile micropipette tips, cells from each colony of interest were added to the PCR mixture as a DNA template. PCR was performed as follows: 95 °C for 5 min, 40 cycles of (95 °C for 30 s, 54 °C for 30 s, 72 °C for 2 min), and finally, 72 °C for 10 min. PCR product was resolved *via* electrophoresis on a 1% agarose gel in 0.5x tris-borate-EDTA buffer. The PCR product of interest was then purified using a QIAquick PCR purification system following the manufacturer's protocol (Qiagen). Sanger sequencing was performed by Microsynth. Sequences were aligned using Geneious software (Biomatters Inc.).

### Generation of point mutations using CRMAGE

Point mutations were generated in *E. coli* genome using a CRMAGE protocol (24) as described earlier (20). In brief, the strain of interest was transformed with a pMA7CR_2.0 plasmid encoding the CRISPR-Cas9 machinery and a λRed recombinase. A second plasmid pMAZ-SK, encoding a gRNA targeting the gene of interest was then electroporated along with an oligonucleotide containing the mutation to insert. Colonies resulting from this electroporation were tested by colony PCR with primers amplifying the region of the gene of interest targeted by mutagenesis and subsequent Sanger sequencing, as described above. All oligonucleotides that were used are listed in Table S9.

### Proteomics analyses

*E. coli* MG1655 and corresponding mutants were grown overnight in LB, diluted to 0.1 OD_600,_ and grown for either 2 hours (for comparison of mutants) or 1 hour before the addition or not of 32 μg/ml TAT-RasGAP_317-326_ for 2 hours (for peptide effect measurement). Samples were then prepared as for Western blot analysis. Samples were then digested following the SP3 method (35) using 50 mg/ml magnetic Sera-Mag Speedbeads (Cytiva). Briefly, samples were diluted with SP3 buffer (2% SDS, 10 mM DTT, 50 mM Tris, pH 7.5) and heated 10 min at 75 °C. Proteins were then alkylated with 30 mM (final) iodoacetamide for 45 min at RT in the dark. Beads were added at a ratio 10:1 (w:w) to samples, and proteins were precipitated on beads with ethanol (final concentration: 60%). After three washes with 80% ethanol, beads were digested in 50 μl of 100 mM ammonium bicarbonate with 0.4 μg of trypsin (Promega). After 1.5 h of incubation at 37 °C, the same amount of trypsin was added to the samples for an additional 1.5 h of incubation. Two sample volumes of isopropanol containing 1% TFA were then added to the digests, and the samples were desalted on a strong cation exchange (SCX) plate (Oasis MCX; Waters Corp) by centrifugation. After washing with isopropanol/1% TFA and 2% acetonitrile/0.1% FA, peptides were eluted either in 150 μl of 40% MeCN, 59% water, 1% (v/v) ammonia (Mutants comparison) or in 200 μl of 80% MeCN, 19% water, 1% (v/v) ammonia (peptide effect) and dried by centrifugal evaporation.

Tryptic peptide mixtures for mutants comparison were injected on a Vanquish Neo nano-HPLC system interfaced *via* a nanospray Flex source to a high-resolution Orbitrap Exploris 480 mass spectrometer (Thermo Fisher). Peptides were loaded onto a trapping microcolumn PepMap100 C18 (5 mm × 1.0 mm ID, 5 μm, Thermo Fisher) before separation on a C18 custom packed column (75 μm ID × 45 cm, 1.8 μm particles, Reprosil Pur, Dr Maisch, Germany), using a gradient from 2 to 80% acetonitrile in 0.1% formic acid for peptide separation (total time: 130 min). Data-dependent LC-MS/MS analyses of samples from peptide-treated samples were carried out on a Fusion Tribrid Orbitrap mass spectrometer (Thermo Fisher Scientific) interfaced through a nano-electrospray ion source to an Ultimate 3000 RSLCnano HPLC system (Dionex). Peptides were separated on a reversed-phase custom-packed 45 cm C18 column (75 μm ID, 100 Å, Reprosil Pur 1.9 μm particles, Dr Maisch) with a 4 to 90% acetonitrile gradient in 0.1% formic acid (total time 140 min). Full MS survey scans were performed at 120,000 resolution. A data-dependent acquisition method controlled by Xcalibur software (Thermo Fisher Scientific) was used that optimized the number of precursors selected (“top speed”) of charge 2^+^ to 5^+^ while maintaining a fixed scan cycle of 0.6 s (mutants analysis), resp. 2 s (peptide treatment analysis). Peptides were fragmented by higher energy collision dissociation (HCD) with a normalized energy of 30 to 32% at 15′000 resolution. The window for precursor isolation was of 1.6 m/z units around the precursor and selected fragments were excluded for 60s from further analysis.

Data files were analyzed with MaxQuant 2.5.1 using the option “Match between runs” (mutants analysis), or 2.4.11 (36) incorporating the Andromeda search engine (37) (peptide treatment analysis). Cysteine carbamidomethylation was selected as fixed modification while methionine oxidation and protein N-terminal acetylation were specified as variable modifications. The sequence databases used for searching were the *E. coli* (strain K12) reference proteome based on the UniProt database (version of February 14th, 2024, containing 4′404 sequences), and a “contaminant” database containing the most usual environmental contaminants and enzymes used for digestion (keratins, trypsin, etc). Mass tolerance was 4.5 ppm on precursors (after recalibration) and 20 ppm, resp. 0.5 Da on MS/MS fragments. Both peptide and protein identifications were filtered at 1% FDR relative to hits against a decoy database built by reversing protein sequences.

From the MaxQuant proteinGroups.txt table, contaminant proteins and reverse hits were removed, and intensity iBAQ values (38) were log2-transformed. After assignment to groups, only proteins quantified in at least two samples of one group were kept. After normalization by median subtraction and missing values imputation (by random numbers drawn from a normal distribution), *t* test was carried out between both conditions (*p*-value threshold <0.05). The difference of means obtained from the *t* test was used for 1D enrichment analysis on associated GO/KEGG annotations as described (39). The enrichment analysis was also FDR-filtered (Benjamini-Hochberg, Q-val<0.02).

### Growth curves and viability test

Overnight cultures were diluted to 0.01 OD_600_ in fresh LB and incubated at 37 °C under constant shaking. Growth was monitored by OD_600_ measurement at the indicated time points either manually or using a 96-well plate reader a BioTek, EPOCH 2 microplate spectrophotometer (Agilent).

The viability of *E. coli* strains in the presence of antimicrobial agents was tested by CFU measurement. Overnight cultures were diluted to 0.1 OD_600_ and grown for 1 hour at 37 °C under constant shaking. Antimicrobial agents were then added, and samples of the culture were harvested at the indicated time points. Serial ten-fold dilutions of bacterial cultures were performed and 10 μl of each dilution were spread onto LB agar plates and incubated overnight at 37 °C. Numbers of colonies were counted for the dilutions for which separated colonies could be observed and CFU/ml values were calculated.

### Microscopy

Overnight cultures were diluted to 0.1 OD_600_ and grown for 1 hour at 37 °C under shaking. Drugs were added when indicated and incubation was continued for two more hours. Then, 500 μl of culture was harvested by centrifugation at 5000 rpm for 2 min. Next, 450 μl of the supernatant were discarded and the pellet was resuspended in the remaining medium. Finally, 5 μl of the bacterial suspension were placed in between slide and coverslip and observed under a Axioplan two microscope with a 100x oil immersion objective (Zeiss). Images were taken with ZenBlue 3.2 software and treated with Fiji software (40). The cell area was quantified using Fiji software using the lasso tool to outline the bacteria.

### SDS-PAGE and Western blot

The density of cultures of the strains of interest, treated or not with antimicrobial agents, was measured. A quantity of culture corresponding to 0.2 OD_600_ was harvested, centrifuged at full speed for 5 min, and resuspended in 100 μl of Laemmli buffer. 10 μl of the suspension were loaded on a 10% polyacrylamide gel and migration was performed for 45 min at 35 mA per gel. Proteins were then transferred on a nitrocellulose membrane by electroblotting at 75V for 1h. The membrane was incubated with Ponceau S, washed with double-distilled water, and then, an image was taken. The membrane was blocked with 5% milk in saturation buffer for 2h and then incubated in primary antibody (2500x diluted in 0.5% milk) overnight. After three washes in 0.5% milk, the membrane was incubated 2h in a 2500x dilution of HRP-conjugated goat anti-rabbit antibody (Promega). After three washes with saturation buffer, the membrane was incubated for 2 minutes with revelation kit and chemiluminescence was measured using a Fusion FX6 (Vilber). Images were treated and band intensity was quantified using ImageJ (41).

### *In silico* modeling

Interactions between the β-barrel subunit of BamA protein from *E. coli* K12 in closed inward position (PDB ID: 5D0O) (40) and TAT-RasGAP_317-326_ (DTRLNTVWMWGGRRRQRRKKRG) peptide in the retro-inverso configuration were studied using molecular dynamics with the initial interacting structures obtained from a molecular docking protocol. The docking of TAT-RasGAP_317-326_ (TRG) with BamA surrounded by a lipid bilayer was performed with HADDOCK 2.4 (42), using as receptor target the BamA loop 3 (from Q495 to T505) and as ligand the whole TAT-RasGAP_317-326_ peptide allowing a full flexibility of the RasGAP_317-326_ moiety. Docking was performed on wild-type version of BamA, as well as mutants A499 V, K798D, D498 N, and D497N-D498K-D500 N (3MUT-N). The first 10 docking poses for all five systems were used as starting positions for the molecular dynamics simulations. The membrane was composed of 200 phospholipids in total, including 114 PYPE (phosphatidylethanolamine - 16:0/16:1), 30 POPE (phosphatidylethanolamine - 16:0/18:1), 16 OYPE (phosphatidylethanolamine - 18:1/16:1) and 40 POPG (phosphatidylglycerol - 16:0/18:1) as previously done (43), constructed and solvated according to the TIP3P water model using CHARMM-GUI (44). All the systems were composed of around 95′000 particles, after the addition of sodium and chloride ions at a concentration of 0.15 M. The CHARMM36 (45) force field was used to define phospholipids and protein topology through an all-atom approach. Each system was minimized using the steepest descent method. We then performed the equilibration procedure through one molecular dynamics simulation of 250 ps under the NVT ensemble and four MD simulations of 250 ps, 500 ps, 1 ns, and 5 ns under the NPT ensemble. Phospholipids position restraints were applied during the first five MD equilibrations and gradually removed from 1000 kJ/mol·nm2 to 0 kJ/mol·nm2. Protein position restraints were also gradually removed from 4000 kJ/mol·nm2 to 50 kJ/mol·nm2. For the equilibration protocol, the velocity rescaling (46) temperature coupling algorithm with a time constant of 1.0 ps was applied to keep the temperature at 310 K. c-rescale semi-isotropic pressure coupling algorithm (47) with reference pressure of 1 bar and a time constant of 5.0 ps was employed. Then, all systems were simulated for the production run in the NPT ensemble with 2 fs time steps, using a v-rescale thermostat and c-rescale barostat, with a time constant of 1.0 ps and 5.0 ps, respectively. Each starting docking pose was simulated for 200 ns in three replicas, for a total simulated time of 30 μs. Electrostatic interactions were calculated by applying the particle-mesh Ewald (PME) method and van der Waals interactions were defined within a cut-off of 1.2 nm. Periodic boundary conditions were applied in all directions. Trajectories were collected every 2 ps and the Visual Molecular Dynamics (VMD) (48) package was employed to visually inspect the simulated systems. GROMACS 2023 (49) package was used for simulations and data analysis. The last 20 ns of the 200 ns production run of each simulation were considered for the analyses.

### Checkerboard assays

Checkerboard assays were performed as previously described (21). Shortly, the bacterial inoculum was prepared for MIC measurement. Two-fold serial dilutions of MRL-494 and TAT-RasGAP_317-326_ were made across the rows and columns of a 96-well plate in a final volume of 100 μl of LB medium. 10 μl of prepared bacterial inoculum was added to each well and the plate was incubated statically at 37°C for approximately 16 h. Growth was measured as described for MIC measurement. The fractional inhibitory concentration index was calculated with the formula detailed elsewhere (21).

## Data availability

The proteomic dataset can be found in supporting information. All raw MS data together with raw output tables are available *via* the Proteomexchange data repository (www.proteomexchange.org) with the accession numbers PXD055766 and PXD051962. Other raw data will be made available upon request to the corresponding author.

## Supporting information

This article contains supporting information.

## Conflict of interest

The authors declare that they have no conflicts of interest with the contents of this article.
