# Supplementary material for: Mutations in the essential outer membrane protein BamA contribute to Escherichia coli resistance to the antimicrobial peptide TAT-RasGAP317-326
Source: J Biol Chem. 2024 Nov 26;301(1):108018. doi: 10.1016/j.jbc.2024.108018 (PMC11842939; doi:10.1016/j.jbc.2024.108018)

Figure S1. EnvZ^{D233N} mutation causes a decrease in bacterial cell size. The indicated mutants were grown overnight and diluted to 0.1 OD_{600}. After two hours of growth at 37°C, bacteria were observed by microscopy. Cell area of 100 cells was measured using Fiji program. Black lines represent means and P values are shown for EnvZ^{D233N} mutants in comparison with their parental counterpart.

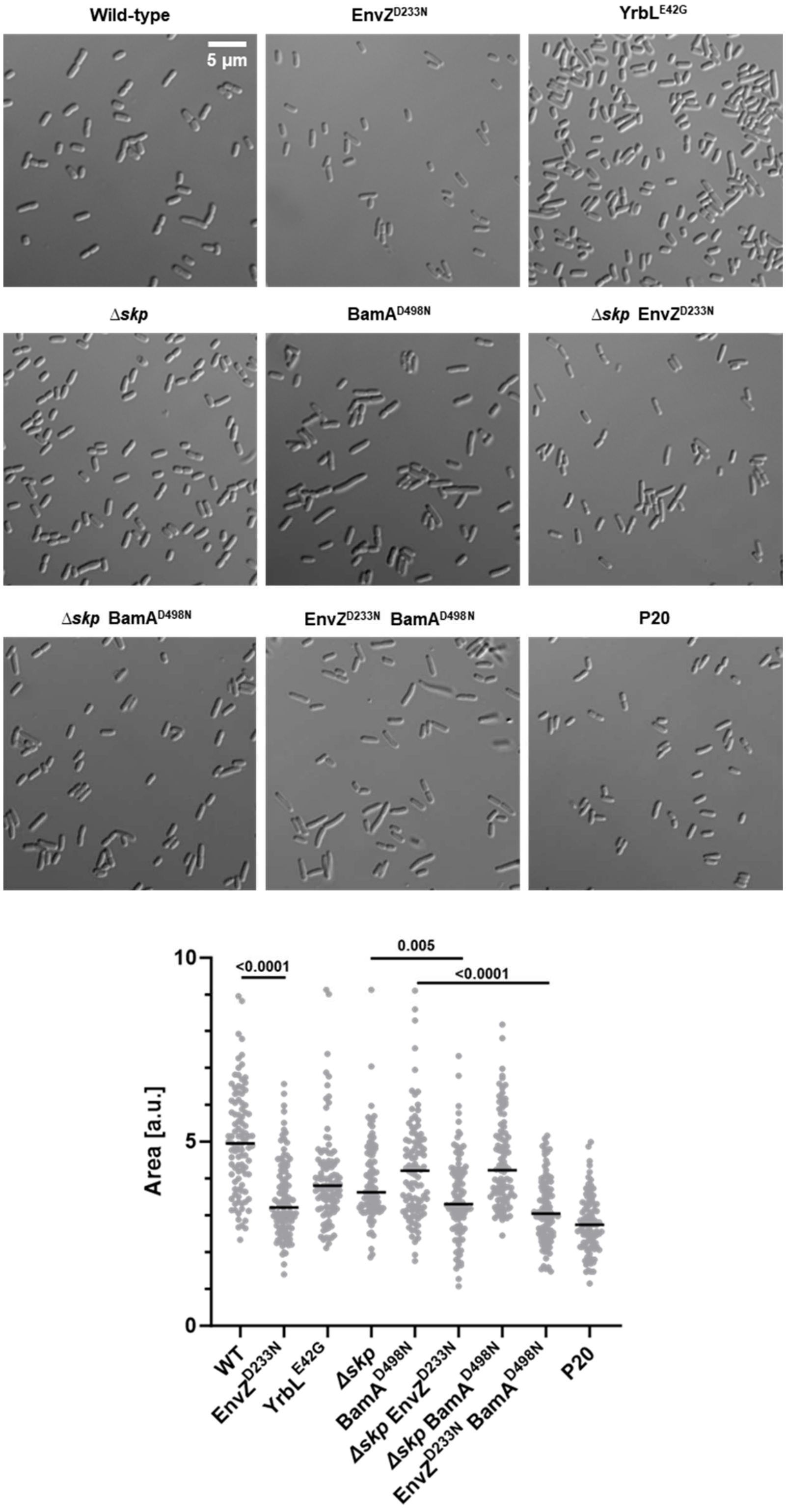

Figure S2. EnvZ^{D233N} mutations causes a moderate growth defect in vitro, while *skp* deletion increases growth rate. Overnight cultures of the indicated strains were diluted to 0.01 OD_{600} and growth in LB at 37°C under constant shaking was monitored each 30 minutes for a total of 24h. Error bars show standard deviation of three independent experiments.

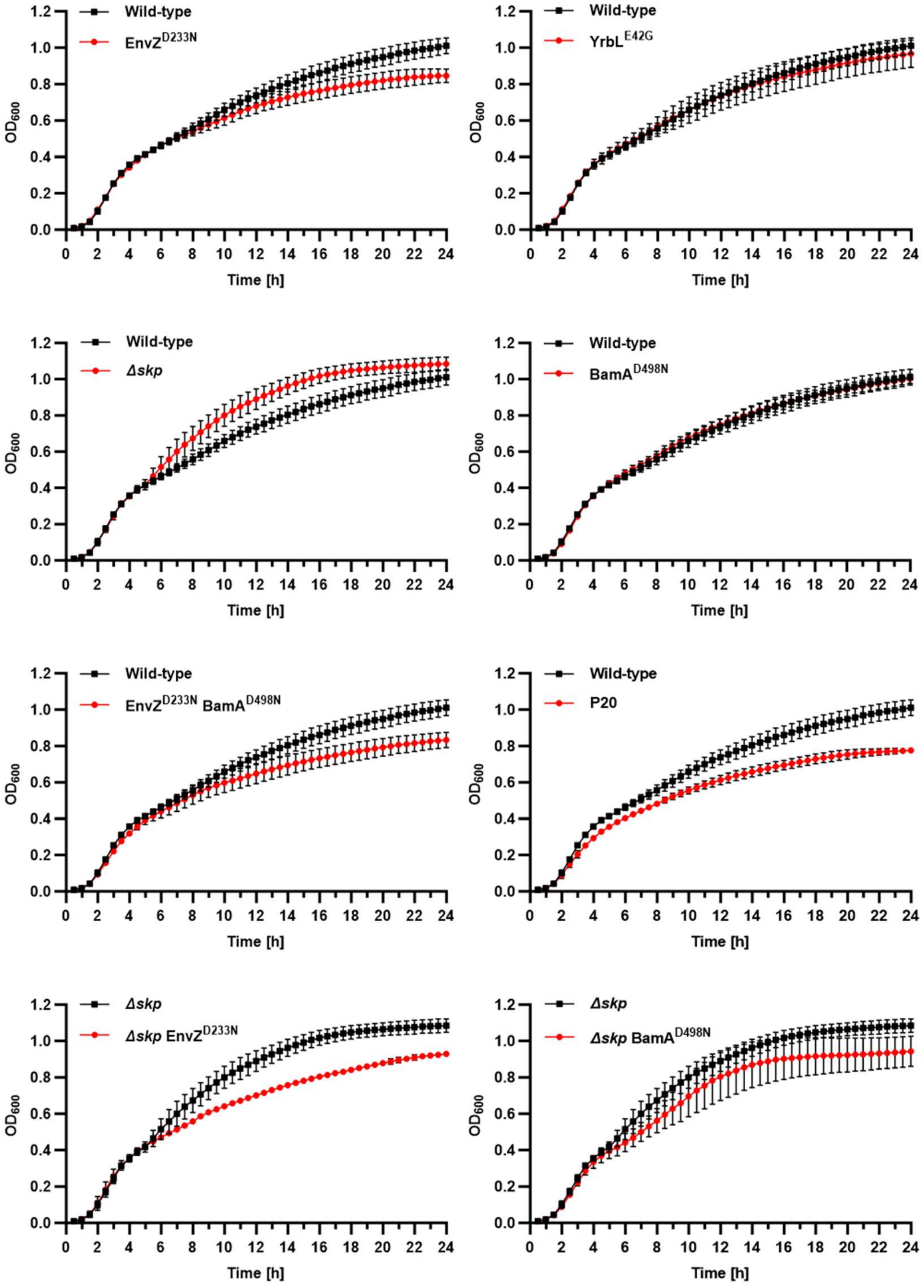

Figure S3. BamA point mutants used in this study show no growth defects. Indicated strains were grown overnight and diluted to OD_{600} = 0.01. Growth was monitored by OD_{600} measurement at the indicated time points. Error bars show standard deviation for two independent replicates.

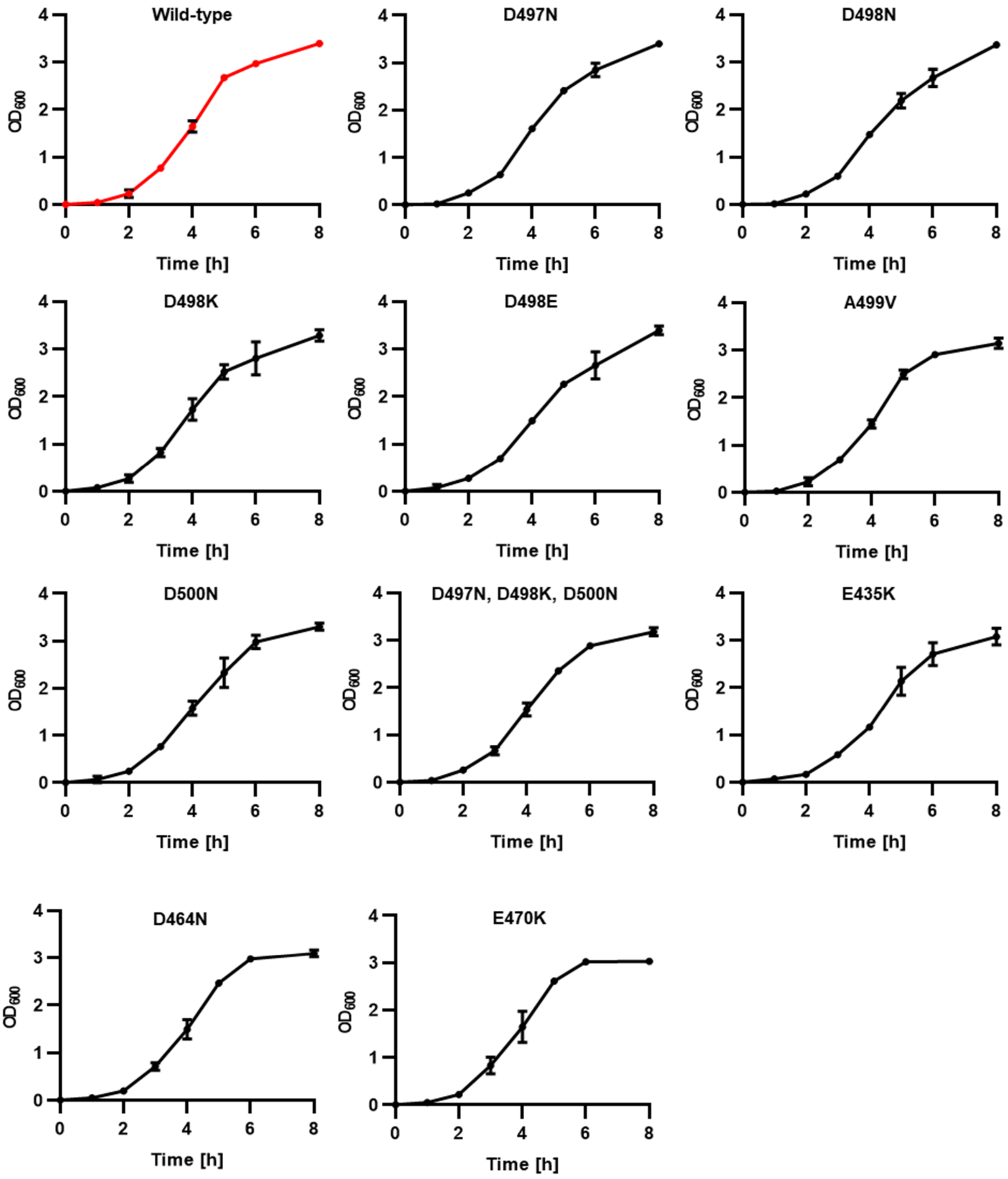

Figure S4. BamA point mutants used in this study show no defects in bacterial morphology. Indicated strains were grown overnight, diluted to OD_{600} = 0.1 and incubated for two hours. Pictures were taken using brightfield microscopy.

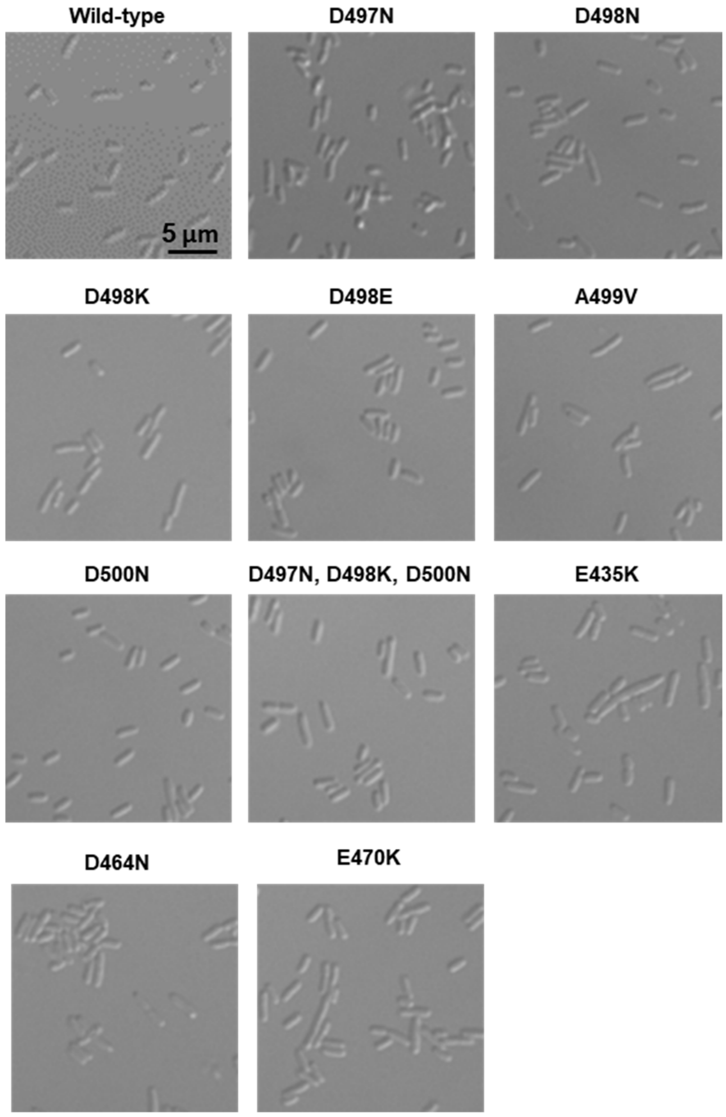

Figure S5. BamA point mutations are not associated with changes in BamA protein levels and activity. BamA point mutations do not influence BamA protein level and activity. Overnight cultures of the indicated mutants were diluted to OD_{600} = 0.1 and grown for two hours. Equivalent quantities of bacteria (normalized by OD_{600} measurement) were harvested and levels of BamA **(A)** and OmpC **(B)** were quantified by Western blotting in duplicate. "Triple mut" corresponds to the $BamA^{D497N,\ D498K,\ D500N}$ mutant. Analysis was performed using ImageJ and intensity of the bands was normalized using $BamA^{WT}$ strain as a control.

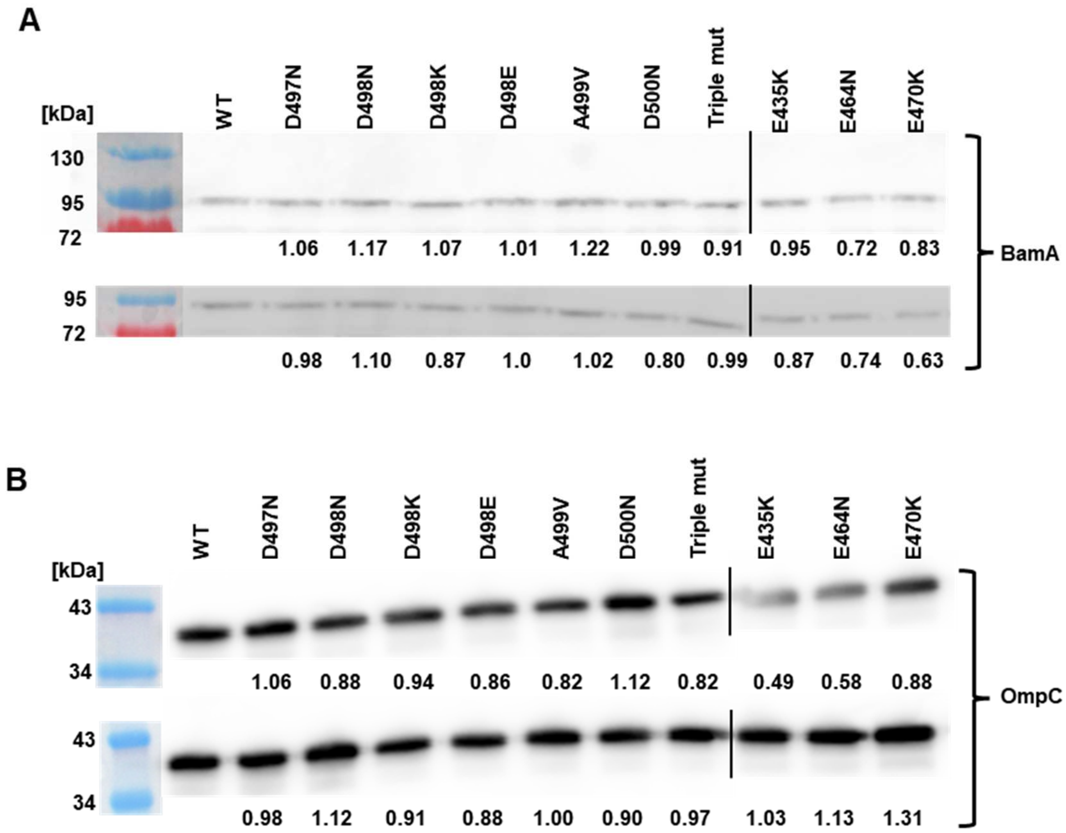

Figure S6. EnvZ is required for BamA mutants to increase TAT-RasGAP$_{317-326}$ MIC (A) and survival to this peptide (B). Experiments were performed as detailed in Figure 3 B and C, but in an *envZ* deletion mutant background.

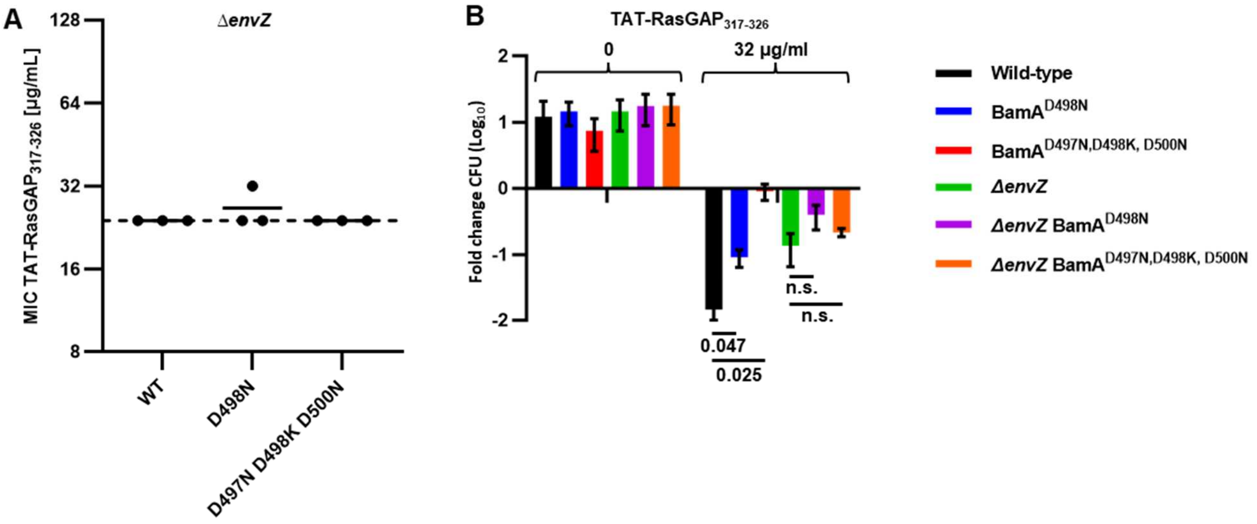

Figure S7. Deletion of non-essential components of the Bam complex impacts its activity. Equivalent quantities of bacterial extracts from indicated strains were used to perform Western blotting with anti-BamA and anti-OmpC antibodies. Ponceau staining was performed to control that equivalent protein amounts were loaded in each well. Levels of BamA and OmpC were quantified using ImageJ and intensity of the bands was normalized using the wild-type strain as a control. The experiment was performed in duplicates. A representative experiment and average and standard deviations of the two replicates are shown.

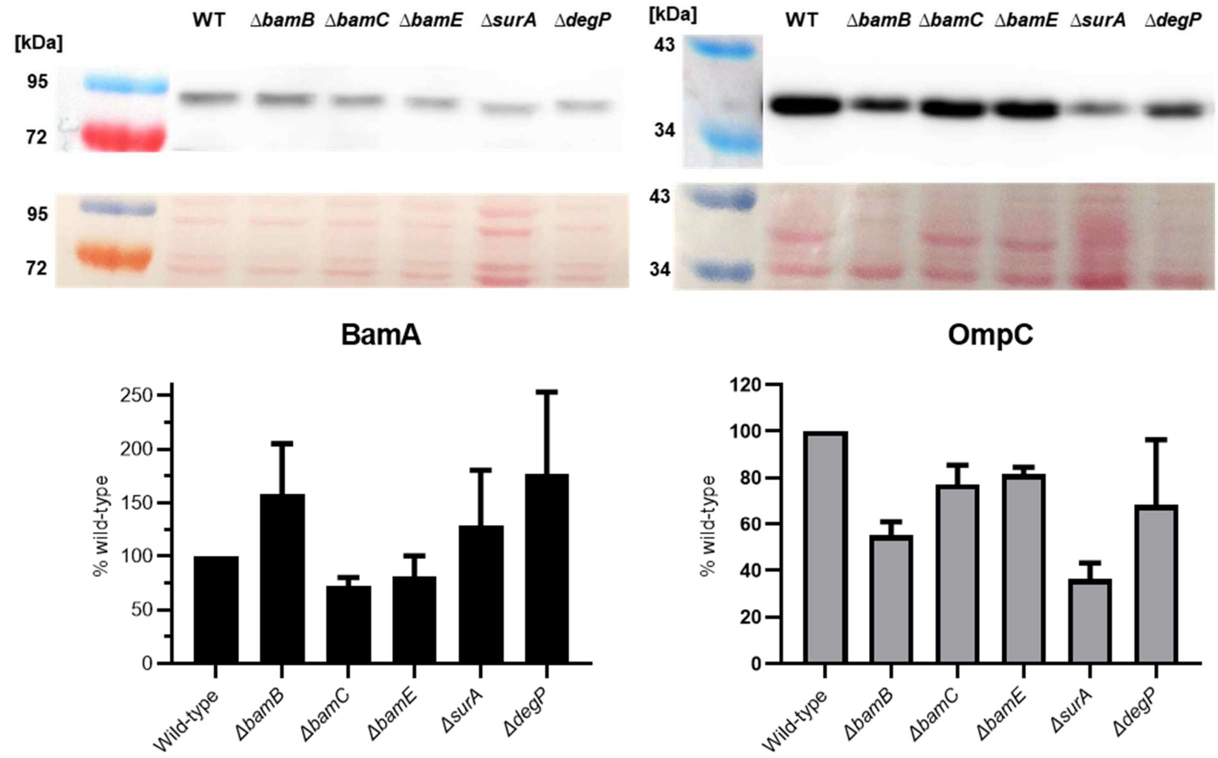

Figure S8. Effect of different concentrations and different times of incubation on BamA and OmpC protein levels. Bacteria were treated as for Figure 8C. Western blotting was then performed with anti-BamA **(A)** or anti-OmpC **(B)** antibodies and protein levels were quantified as for Figure 8A.

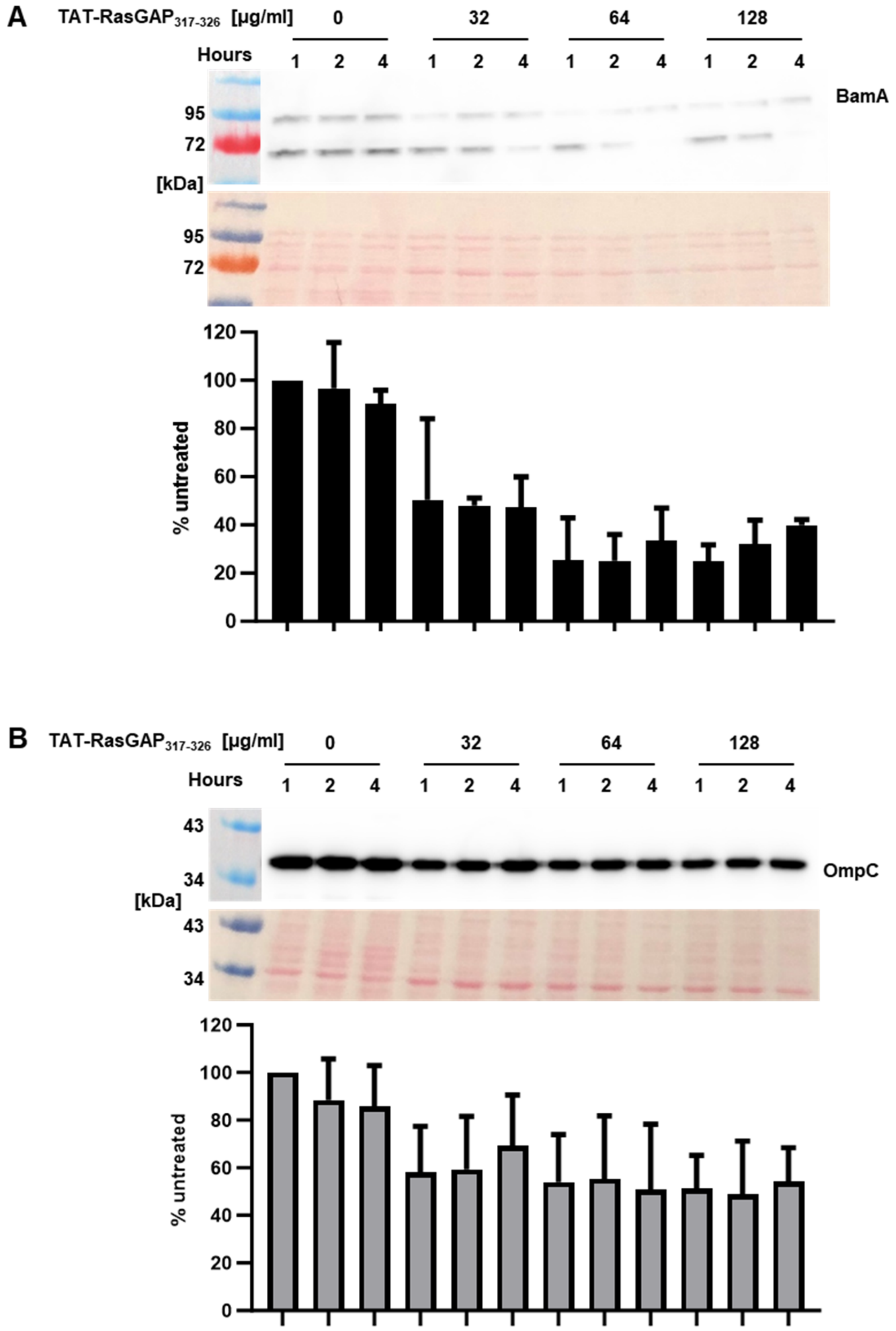

Figure S9. TAT-RasGAP$_{317-326}$ and BamA inhibitors have different effects on *E. coli* morphology. Wild-type *E. coli* strain was grown overnight, diluted to OD_{600} = 0.1 and incubated for one hour at 37°C with shaking. Bacteria were then treated with the indicated concentrations of drugs for one hour and pictures were taken by brightfield microscopy.

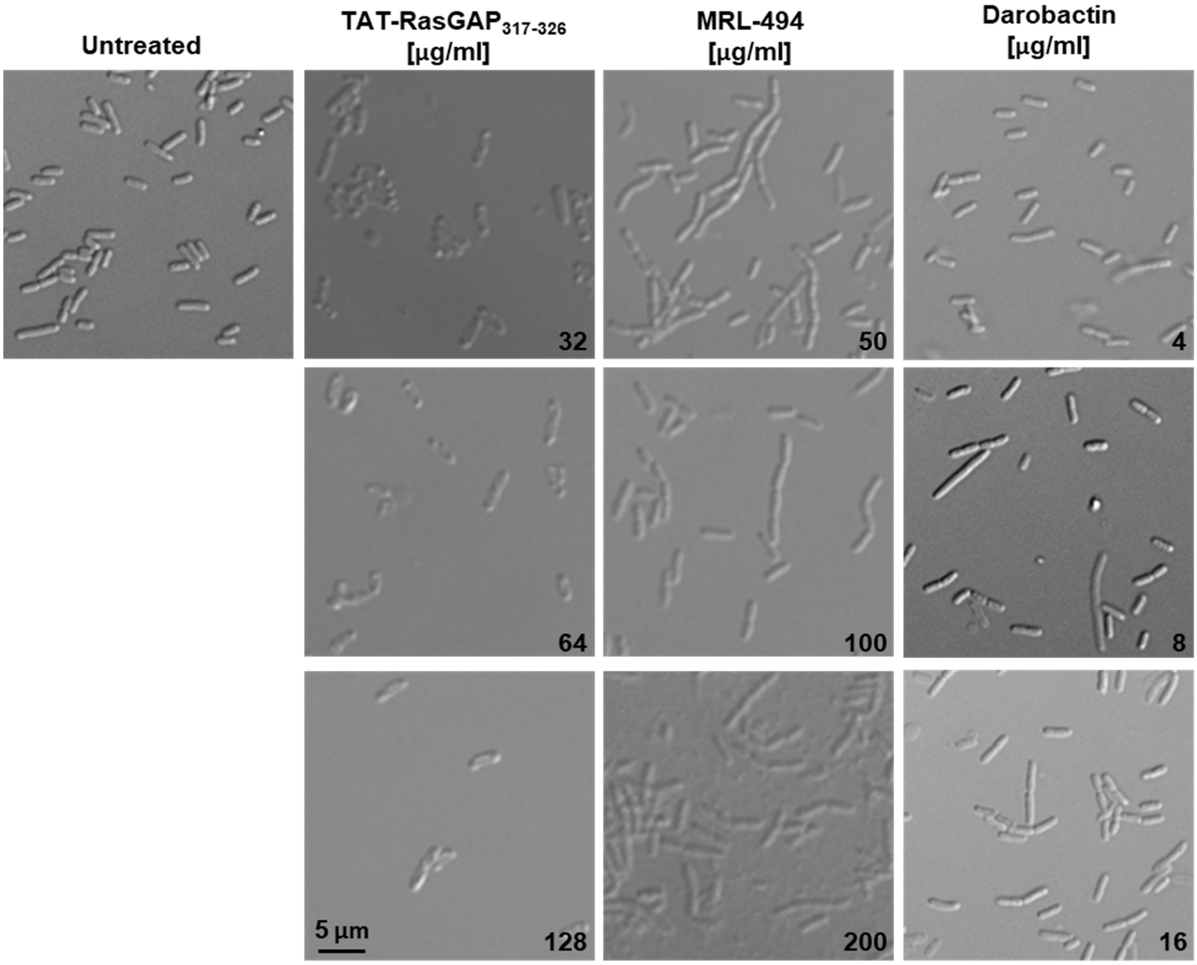

Supplement: Supplementary Figures [file mmc1.pdf]
